# Are asymmetric inheritance systems an evolutionary trap? Transitions in the mechanism of paternal genome loss in the scale insect family Eriococcidae

**DOI:** 10.1093/genetics/iyad090

**Published:** 2023-05-15

**Authors:** Christina N Hodson, Alicia Toon, Lyn G Cook, Laura Ross

**Affiliations:** Institute of Evolutionary Biology, University of Edinburgh, Edinburgh, EH9 3JT, UK; Department of Zoology, Biodiversity Research Centre, University of British Columbia, Vancouver, BC, V6T 1Z4, Canada; School of Biological Sciences, University of Queensland, Brisbane, QLD 4072, Australia; School of Biological Sciences, University of Queensland, Brisbane, QLD 4072, Australia; Institute of Evolutionary Biology, University of Edinburgh, Edinburgh, EH9 3JT, UK

**Keywords:** epigenetic modification, heterochromatization, paternal genome elimination, genomic conflict, uniparental transmission

## Abstract

Haplodiploidy and paternal genome elimination (PGE) are examples of asymmetric inheritance, where males transmit only maternally inherited chromosomes to their offspring. Under haplodiploidy, this results from males being haploid, whereas under PGE, males inherit but subsequently exclude paternally inherited chromosomes from sperm. Their evolution involves changes in the mechanisms of meiosis and sex determination and sometimes also dosage compensation. As a result, these systems are thought to be an evolutionary trap, meaning that once asymmetric chromosome transmission evolves, it is difficult to transition back to typical Mendelian transmission. We assess whether there is evidence for this idea in the scale insect family Eriococcidae, a lineage with PGE and the only clade with a suggestion that asymmetric inheritance has transitioned back to Mendelian inheritance. We conduct a cytological survey of 13 eriococcid species, and a cytological, genetic, and gene expression analysis of species in the genus *Cystococcus*, to investigate whether there is evidence for species in this family evolving Mendelian chromosome transmission. Although we find that all species we examined exhibit PGE, the mechanism is extremely variable within Eriococcidae. Within *Cystococcus*, in fact, we uncover a previously undiscovered type of PGE in scale insects that acts exclusively in meiosis, where paternally inherited chromosomes in males are present, uncondensed, and expressed in somatic cells but eliminated prior to meiosis. Broadly, we fail to find evidence for a reversion from PGE to Mendelian inheritance in Eriococcidae, supporting the idea that asymmetric inheritance systems such as PGE may be an evolutionary trap.

## Introduction

Reproductive systems (i.e. chromosome inheritance and sex determination) are extraordinarily variable across the tree of life. Understanding the forces that have led to this variety and what leads to or constrains transitions to new systems has been a major goal in evolutionary biology in recent years (Bachtrog *et al.* 2014). In most species, chromosome inheritance is a fair process overall such that either homologous chromosome in a diploid has an equal probability of being transmitted to offspring (i.e. Mendelian chromosome transmission). However, some reproductive systems are characterized by unequal chromosome transmission (asymmetric inheritance or non-Mendelian transmission). Haplodiploidy and paternal genome elimination (PGE) are the 2 most widespread examples of these (de la Filia *et al.* 2015; Ross *et al.* 2022). In both haplodiploidy and PGE, males transmit only maternally derived chromosomes to their offspring. However, while under haplodiploidy, males develop from unfertilized eggs; in PGE, males develop from fertilized eggs and are diploid, but paternally inherited chromosomes are not incorporated into viable sperm (Normark 2003). It remains unclear how haplodiploidy and PGE evolve, yet these systems appear to be extraordinarily successful and have evolved repeatedly, with more than 20 origins across invertebrates. They are found across large clades, including entire orders (e.g. Hymenoptera, thrips, and globular springtails), and make up around 12% of extant animal species (de la Filia *et al.* 2015).

Ideas about the evolution of haplodiploidy and PGE suggest that genomic conflict, specifically conflict between the maternal and paternal halves of the genome, drives transitions to these systems (Hartl and Brown 1970; Bull 1979, 1983; Burt and Trivers 2006; Gardner and Ross 2014; Ross *et al.* 2019). In these models, asymmetric transmission benefits maternally derived chromosomes/genes in males as they are transmitted to all male offspring (rather than 50%, as expected under Mendelian transmission). Therefore, we might expect the evolution of asymmetric inheritance to be quite dynamic, especially in early stages, with frequent transitions between Mendelian and non-Mendelian inheritance, depending on which party in conflict gains the upper hand. An indication of this conflict would be species with Mendelian inheritance within clades with asymmetric inheritance. Currently, there is not a single record of transitions from haplodiploidy back to Mendelian inheritance, and the evidence for transitions within PGE clades remains unconfirmed. Transitions from Mendelian to asymmetric inheritance involve a number of changes. Changes in the mechanism of meiosis, sex determination, ploidy, and dosage compensation, among others, often occur with a shift to asymmetric inheritance (Gardner and Ross 2014; Ross *et al.* 2019). Because of the number and complexity of these changes, asymmetric inheritance systems are thought to be an evolutionary trap (i.e. once they evolve, it may be difficult to transition back to Mendelian inheritance) (Bull 1983; Bachtrog *et al.* 2014). The apparent absence of transitions from haplodiploidy to diplodiploidy—or more precisely a lack of diploid species within haplodiploid clades (Tree of Sex database, http://www.treeofsex.org/)—has been taken as evidence for such an evolutionary trap (Bull 1983; Bachtrog *et al.* 2014; Blackmon *et al.* 2017). It is less clear if PGE is a similar evolutionary trap or if reversions to Mendelian reproduction are possible.

Males in species with PGE, unlike those with haplodiploidy, are often diploid with paternal chromosomes eliminated only in germline cells undergoing meiosis (de la Filia *et al*. 2015). So, transitions back to diplodiploidy may not involve a change in male ploidy, and spermatogenesis often still involves 2 meiotic divisions. However, the evolution of PGE does involve significant changes in the mechanism of meiosis such that chromosomes segregate according to their parent of origin and, in many species with PGE, meiosis is also altered in other ways. For instance, in scale insects, the meiotic sequence is reversed (i.e. inverted meiosis), and in both scale insects and fly lineages with PGE, a highly derived monopolar spindle is present in male meiosis, which only attaches to maternally inherited chromosomes (Brown 1967; Kubai 1982; Bongiorni *et al.* 2004). Furthermore, all documented lineages with PGE have evolved from an XO or XY sex determination system, and the evolution of PGE from these sex chromosome systems requires a change in the sex determination mechanism since otherwise males in PGE systems would always transmit the maternally derived X chromosome through sperm, resulting in female-only offspring (Gardner and Ross 2014). Therefore, lineages with PGE have evolved unconventional sex determination systems, either involving elimination of sex chromosomes after fertilization or silencing/elimination of paternally inherited chromosomes in males (Du Bois 1933; Nur 1990; Bongiorni *et al.* 2001). Additionally, although males generally remain diploid, in some PGE systems, the mechanism of PGE has evolved such that some paternally inherited chromosomes are eliminated or transcriptionally repressed in cells of males early in development, and therefore, the evolution of PGE causes males to exhibit haploid rather than diploid gene expression in somatic cells (Brown and Bennett 1957; de la Filia *et al.* 2021).

So, is there any evidence that Mendelian reproduction has re-evolved within PGE clades? PGE has evolved independently at least 7 times, and although it generally occurs across large clades, many of these are poorly studied in terms of both biology and systematics (Gardner and Ross 2014; de la Filia *et al.* 2015). The only clade for which a considerable number of taxa (around 500 or ∼5% of described species) have been studied is the scale insect (Hemiptera: Coccoidea) (Nur 1980, p. 19; Gavrilov 2007; Ross *et al.* 2010). The phylogenetic distribution of PGE across this clade suggests that transitions back to Mendelian chromosome inheritance are rare but have potentially occurred. In mealybugs, there is evidence that some paternal chromosomes occasionally escape elimination during meiosis (de la Filia *et al.* 2019). Additionally, in 2 scale insect species, *Stictococcus* sp. and *Lachnodius eucalypti*, cytogenetic analyses suggest that PGE may be absent (Brown 1977; Nur 1980). These 2 species are relatively closely related and belong to a scale insect family with substantial variability in the mechanism of PGE (Eriococcidae sensu lato). This variability has been argued to be due to an evolutionary arms race between maternal and paternal alleles over paternal transmission in males, suggesting that scale insects, and in particular, the family in which *Stictococcus* sp. and *L. eucalypti* belong, should be investigated in more detail, as this may be a group in which conflict over chromosome transmission to future generations is high (Brown 1964; Herrick and Seger 1999; Ross *et al.* 2010).

Here, we focus on scale insects within the family Eriococcidae and explore whether there is evidence for paternally inherited chromosomes regaining expression/transmission through males. The Eriococcidae belong to Neococcoidea, a large monophyletic clade (14 families, 6,000 species) of scale insects that have evolved PGE (Ross *et al.* 2010). Although males across Neococcoidea do not transmit paternally inherited chromosomes through sperm, there is variability in the mechanism of PGE between species, both during meiosis and during early embryogenesis (Supplementary Fig. 1). The differences in the mechanism of PGE are classified into several categories within scale insects, which differ in whether, in somatic cells, paternally inherited chromosomes are retained and heterochromatized (epigenetically silenced) in embryogenesis or whether they are eliminated entirely in embryogenesis. In both cases, males have limited or no expression of paternal chromosomes in somatic cells (de la Filia *et al.* 2021). Additionally, during meiosis, the categories differ in whether there is 1 division in meiosis or 2 and whether any paternally inherited chromosomes are eliminated prior to meiosis (Nur 1980; Ross *et al.* 2010).

Eriococcid scale insects exhibit a system known as Comstockiella PGE, where in males, paternally inherited chromosomes are condensed into a heterochromatic body (a clump of facultatively heterochromatized chromosomes on the edge of the nucleus) in somatic cells, and during meiosis, some paternally derived chromosomes are eliminated prior to meiotic divisions, with any remaining paternally derived chromosomes segregating into pycnotic nuclei, which do not form into viable sperm. This is thought to be the ancestral reproduction system in this family, but there is remarkable diversity in how meiosis occurs in different species (Gavrilov 2007; Ross *et al.* 2010). For instance, the number of paternal chromosomes eliminated in males prior to meiosis differs between and even sometimes within species, leading to differences in the number of divisions in meiosis and differences in whether pycnotic nuclei form after meiosis (Brown 1967; Nur 1980). For both of the suggested losses of PGE in Eriococcidae, in *L. eucalypti* and *Stictococcus* sp., there is no clear evidence that paternal chromosomes are eliminated in male meiosis and—unlike in other scale insects with PGE—somatic cells in males lack heterochromatic bodies containing the silenced paternal genome. This led researchers to suggest that PGE was absent in these species (Brown 1967; Nur 1980). However, this conclusion was based on cytological observations of a small number of specimens, and both of these species have uncertain phylogenetic placements, with *L. eucalypti* thought to belong to the Gondwanan clade of eriococcid scale insects and *Stictococcus* sp. now potentially placed in Stictococcidae, which is nested inside Eriococcidae sensu lato (Cook *et al.* 2002; Cook and Gullan 2004; Gullan and Cook 2007). A more thorough examination, encompassing genetic as well as cytological data, is needed to understand whether and how transitions to Mendelian inheritance in Eriococcidae have occurred and whether transitions have occurred once or several times.

We investigate whether there is evidence for transitions from PGE to Mendelian inheritance in species within Eriococcidae using cytological, genetic, and transcriptomic analyses. In eriococcid species that exhibit Comstockiella PGE, somatic cells of males have heterochromatic bodies containing paternal chromosomes. We therefore first examined somatic tissue in 13 eriococcid species to determine whether heterochromatic bodies are present in somatic cells of males. We then investigate male meiosis in a subset of species, focusing on the *Ascelis*/*Cystococcus* clade, which shows variation in whether heterochromatic bodies are visible. We study the inheritance patterns (using microsatellite markers) in 2 species of *Cystococcus*: *Cystococcus campanidorsalis*, in which male heterochromatic bodies are present, and *Cystococcus echiniformis*, in which male heterochromatic bodies are absent. This allowed us to determine whether inheritance patterns from mothers to sons are consistent with what we would expect under PGE inheritance. Finally, we generate RNA-seq data from females and 2 offspring for *C. echiniformis* and *C. campanidorsalis* families to determine whether a loss of heterochromatic bodies corresponds to a transition in gene expression profiles such that paternally inherited genes are expressed (i.e. diploid rather than haploid expression of chromosomes). We find that although some species within Eriococcidae do not have heterochromatic bodies in somatic tissue in males, there is no evidence that PGE has been lost in these species; rather, there has been a transition to a different type of PGE, unlike those described so far in scale insects. Interestingly, we also find that both *C. campanidorsalis* and *C. echiniformis*, which differ in whether they have heterochromatic bodies, have substantial expression of paternally inherited chromosomes in males. This suggests that this lineage has undergone a change from uniparental to biparental expression in the past and also that the presence of heterochromatic bodies does not always correspond to a complete lack of paternal chromosome expression in males with PGE. More broadly, we find no evidence for a transition from PGE back to Mendelian reproduction in eriococcid scale insects and thus fail to reject the idea that PGE is an evolutionary trap.

## Materials and methods

### Study system

Eriococcidae is a diverse and widespread family of scale insects. We focus primarily on the monophyletic subclade, labeled the Gondwanan clade by Cook and Gullan (2004), which has approximately 70 species, is relatively well characterized, and contains *L. eucalypti* (a species reported to lack PGE). All species are ectoparasites of trees and shrubs in the myrtle family (Myrtaceae). Most species are highly host plant-specific, and many in the clade form galls (Cook and Gullan 2004). Although species within Eriococcidae can vary in the chromosome number from 2n = 4–192 (Cook 2000), the chromosome number in most species in the Gondwanan clade is 2n = 18 where known (Brown 1967; Cook 2000). Because the chromosomes in some species are too numerous and small to count individually, we confined our analyses to the behavior of the maternally vs paternally inherited chromosomes in males.

### Staining of male somatic tissue for the presence of heterochromatic bodies

Samples were collected in Queensland and New South Wales, Australia, from 2010 to 2017 (see Supplementary Table 1 for details) and stored in 1:3 glacial acetic acid:ethanol fixative at 4°C. We performed DAPI staining on somatic tissue of males for 13 species of Eriococcidae to determine whether heterochromatic bodies are present. The majority of species had not previously been studied cytologically, although we also included *Ascelis schraderi* and *Eriococcus coriaceus*, which had previously been examined by Brown (1967), to ensure that our staining techniques were producing comparable results. We did not include any species in the genus *Apiomorpha*, as 45 species within this group have already been examined, all of which were found to have heterochromatic bodies in males (Cook 2000). With the species examined previously and in our study, we now have information about whether somatic heterochromatization is present in 79 species of Eriococcidae.

Many eriococcid species are sexually dimorphic at an early stage, and some exhibit sexual dichronism (i.e. male and female offspring are produced at different times in the female reproductive period) (e.g. *Cystococcus*, Gullan and Cockburn 1986). Thus, it is often possible to sex individuals, even at very early stages of development. We preferentially stained males from later life stages, but when we were unable to determine the sex (embryos or first instar larvae), we stained at least 10 individuals to try to ensure that we stained some males (note that for all samples for which we were unable to determine the sex of individuals, we also stained older males from the same species; Supplementary Table 2). Otherwise, we stained 2–4 males from each species at each sampling location by spreading the body tissue of each male thinly on a slide and staining with DAPI (further details in Supplementary Methods).

Heterochromatic bodies appear as a densely stained body containing paternally derived chromosomes at the periphery of the cell nucleus in somatic cells (Brown and Nelson-Rees 1961; Bongiorni *et al.* 2001). However, in scale insects with PGE, somatic heterochromatization is not always present in all types of somatic cells (Nur 1967), and in some cases, we could not sex individuals before staining. Therefore, we determined whether heterochromatic bodies were present in any somatic cells in each slide and any species in which we were able to identify heterochromatic bodies in somatic tissue we marked as possessing heterochromatic bodies (see Supplementary Table 2 for information on the number of slides/stages and sex of specimens stained for each species).

### Staining of male meiosis in *Cystococcus/Ascelis*

For species within the *Cystococcus/Ascelis* clade, we also performed DAPI staining on tissue undergoing meiosis in males. We stained males at the late third instar larval/early pupal development stages, as we found that this is the stage when meiosis occurs in males. The staining procedure was the same as for somatic tissue, with the exception that when we dissected the individual, we removed as much somatic tissue as possible from the preparation. Meiosis in male scale insects takes place in a cyst, in which the number of nuclei per cyst is generally consistent in each species (Brown 1967). This allowed us to determine which stage of meiosis was occurring by counting the number of nuclei in each sperm cyst. Additionally, if there are 2 divisions in meiosis and PGE, the final products of meiosis are spermatids containing maternally inherited chromosomes that elongate into functional sperm and nonviable pycnotic nuclei containing paternally inherited chromosomes. Therefore, the presence of pycnotic nuclei at the end of meiosis indicates that the species undergoes PGE. We scored slides by evaluating first whether pycnotic nuclei were present after meiosis, then by examining the number of divisions in meiosis. We did this by counting the number of cells in each sperm cyst and the stage of meiosis that was occurring (i.e. prophase, metaphase, anaphase, etc.) and counting how many sperm were present in sperm bundles at the end of meiosis. Then, we were able to determine how many divisions took place in meiosis by evaluating whether the number of sperm was 4× more than the number of nuclei in primary spermatids (2 divisions in meiosis) or 2× more than the number of nuclei in primary spermatids (1 division in meiosis). However, note that we were not able to view all stages of meiosis for all the species of interest (Supplementary Table 2).

### Eriococcidae phylogeny

We estimated a phylogeny with all species for which we stained male somatic tissue, along with some additional species within Eriococcidae that had previously been examined for somatic heterochromatization (Brown 1967) (Supplementary Table 2), using the mitochondrial COI and the nuclear rRNA 18S genes. For both the COI and 18S loci, some of the nucleotide sequences we used were from previously published studies (Cook *et al.* 2002; Cook and Gullan 2004; Gullan and Cook 2007; Semple *et al.* 2015) (see Supplementary Table 3 for accession numbers). For the nucleotide sequences that we generated ourselves, we sequenced an approximately 600-bp region of the small subunit ribosomal RNA gene 18S. We conducted PCR with the 2880/Br primer set (von Dohlen and Moran 1995) and Sanger-sequenced the products in both directions. For the COI locus, we used approximately 510 bp of the 5′ region (COI barcode region) and Sanger-sequenced in both directions with either the mitochondrial primers PCO_F1 (Park *et al.* 2010) and HCO (Folmer *et al.* 1994) (for the majority of species) or the primers CystCOIF and CystCOIR (Semple *et al.* 2015) (for *Cystococcus* and *Ascelis* species) (see Supplementary Table 4 for primer information and thermocycling conditions). We aligned COI and 18S sequences separately in Geneious Prime (2020.2.3) with the MAFFT plugin (v7.450) (Katoh and Standley 2013). For COI sequences, we did a translation alignment using “invertebrate mitochondrial” as the genetic code. We then used IQ-Tree to generate maximum likelihood phylogenies with parameters -alrt 1000 -B 1000, which estimates ultrafast bootstrap values and SH-aLRT values at nodes with 1,000 replicates each and allows IQ-Tree to select the most appropriate substitution model (v2.0.3) (Guindon *et al.* 2010; Kalyaanamoorthy *et al.* 2017; Hoang *et al.* 2018; Minh *et al.* 2020). An ultrafast bootstrap value of ≥95% and an SH-aLRT value of ≥80% indicate high confidence in that clade given the data. We specified the outgroup as *Parasaissetia nigra*, a scale insect species in the family Coccidae (NCBI accession: KY927598.1, KY924795.1). We estimated a phylogeny from the concatenated alignment (SYM + I + G4 substitution model) with both sets of markers. Additionally, we also estimated phylogenies for each marker alone (Supplementary Fig. 2). Although the topology of phylogenies produced with each marker separately differed in some respects from each other, the number of losses of heterochromatic bodies was consistent across phylogenies.

### Microsatellite inheritance assay

To further explore whether paternal chromosomes are transmitted through sperm, we used microsatellite loci to assess transmission in 2 species of *Cystococcus* that differed in the presence of heterochromatic bodies in somatic cells of males (*C. campanidorsalis*, present; *C. echiniformis*, absent). *Cystococcus* females form large galls, and male offspring develop within their mother's gall. Once males mature, the female gives birth to daughters (first instar offspring) that disperse from the gall on the abdomens of their adult brothers (Semple *et al.* 2015). Therefore, it is possible to collect families from the field consisting of the gall, the female within her gall, and her offspring (Gullan and Cockburn 1986; Semple *et al.* 2015).

We determined allele transmission from mothers to sons in 7 families of *C. campanidorsalis* and 13 families of *C. echiniformis* (Supplementary Table 1). Although we could not directly genotype the father/s, we were able to estimate if the genotypic contribution of the father/s was haploid (as expected under PGE) or diploid (as expected under Mendelian inheritance). We designed 9 primer pairs for each species for polymorphic microsatellite loci from whole-genome sequence data, using QDD software (Meglécz *et al.* 2014) to generate primers (Supplementary Table 5; see Supplementary Methods for primer design information). However, 1 primer for *C. campanidorsalis* gave inconsistent results, so we did not analyze data from that locus, giving a total of 8 loci for that species. We extracted 20 μL DNA using the prepGEM insect extraction kit (ZyGEM) from up to 20 male offspring from each family following the manufacturer’s instructions, and a small portion of body wall tissue from the mother, and performed PCR with the Type-it microsatellite PCR kit (Qiagen) (see Supplementary Methods for additional information on the PCR procedure).

We analyzed the microsatellite profiles of each individual in Geneious Prime (2020.2.3) with the microsatellite plugin, manually calling microsatellite peaks. We assessed allele inheritance patterns collectively for all offspring within the brood. For instance, by comparing the maternal genotype to the genotype of all her offspring, we can infer whether the mother was heterozygous or homozygous and transmitted 1 or 2 alleles to her offspring. We can also infer the paternal contribution to the family (i.e. whether the father transmitted 1 or 2 alleles to offspring) by examining the genotype of all offspring and subtracting the maternal genotype. Using the package lme4 in RStudio (v.3.6.3) (Bates *et al.* 2015; R-Core-Team 2020), we analyzed whether broods inherited a different number of alleles from their mother and father and whether the 2 species show different inheritance patterns, using a generalized linear mixed-effects model with a binomial distribution. We were interested in the number of loci that exhibited haploid vs diploid inheritance patterns in each brood. Therefore, we counted the number of microsatellite loci for which each brood inherited 1 allele vs 2 alleles from each parent and combined the count into a vector for the response variable. We included the species and the source of alleles (maternal/paternal) as fixed effects and an observation-level random effect.

As the samples we analyzed were field-collected, we did not have any information about whether females had mated once or multiply. Therefore, in order to determine whether cases where broods had inherited more than 1 paternal allele were caused by the father being diploid and transmitting either allele to his offspring (i.e. no PGE) or by multiple males with PGE and with different genotypes mating with the same female, we examined the allele inheritance patterns for broods that had inherited 2 paternal alleles for 2 or more microsatellite loci. We would expect that as long as the microsatellite loci were not located in close proximity to each other on the chromosome (note that *Cystococcus* species have at least 2n = 18 chromosomes), the allele inheritance profile for different microsatellite loci would be unrelated to each other if the species does not exhibit PGE. However, in the case that the species does exhibit PGE and the female had mated with multiple males, we would expect the inheritance profile for different microsatellite loci to be related, as offspring with the same father would inherit 1 set of alleles, and the offspring with a different father would inherit a different set of alleles. We conducted a *χ*^2^ test in RStudio, with the number of individuals with each genotype as the observed frequencies and the expectation that allele inheritance is random to determine if the offspring genotypes deviated from what would be expected if individuals in a family had 1 father that did not exhibit PGE.

### Expression of paternally derived alleles in males

Male mealybugs with heterochromatic bodies exhibit suppressed expression of paternally inherited chromosomes in somatic tissue (de la Filia *et al.* 2021). To determine whether somatic cells in *Cystococcus* males without heterochromatic bodies express genes inherited from their father (i.e. exhibit diploid rather than haploid expression), we conducted an RNA-seq analysis to examine allele expression in males and their mother and compare whether males exhibit uniparental (i.e. homozygous) expression of genes on a genome-wide scale. We collected data from males of *C. campanidorsalis*, which have clear heterochromatic bodies and for which we expected expression from primarily maternally inherited genes, and males of *C. echiniformis*, which lack heterochromatic bodies and for which we therefore expected to show diploid (biparental) expression (see Fig. 2 for images of somatic cells). We collected galls for *C. echiniformis* and *C. campanidorsalis* from Queensland, Australia, in 2017 and preserved a small portion of somatic tissue from the female in each gall and the whole body of 2 of her sons in a small volume of RNAlater for extraction. We were able to collect 1 family for *C. campanidorsalis* and 3 families for *C. echiniformis* (Supplementary Table 1).

We extracted RNA from the body wall of females using a TRIzol:chloroform extraction and digesting residual DNA after extraction using DNAse I. Because the male samples were much smaller than the female tissue, we used a different RNA extraction procedure to account for the low yield from these samples. For males, we extracted RNA from the whole body using a modified TRIzol RNA extraction procedure with the Purelink RNA Purification Kit (Promega), followed by amplification of the product with the Ovation RNA-seq System V2 (Tecan) (see Supplementary Methods for full RNA extraction protocols). The samples were sequenced by Edinburgh Genomics, generating 150-bp paired-end reads with TruSeq stranded mRNA-seq (or DNA) libraries, as appropriate.

We trimmed the sequence with fastp with settings –cut_by_quality 5 –cut_by_quality 3 –cut_window_size 4 –cut_mean_quality 20 (v0.12.3) (Chen *et al.* 2018), then assembled a de novo transcriptome for both species with all libraries for that species using Trinity with parameters –full_cleanup and –SS_lib_type RF (v2.8.4) (Grabherr *et al.* 2011). We then conducted a BUSCO analysis (v3.0.2) (Waterhouse *et al.* 2018) on each species using the insecta_odb9 database (Supplementary Table 6). As de novo transcriptome assemblies can result in an unexpectedly high number of predicted transcripts, we used previously established approaches to filter the number of transcripts based on expression (Moghadam *et al.* 2013). We mapped the trimmed reads of each species to the appropriate transcriptome using RSEM with parameters –paired-end –bowtie2 (v1.3.1) (Li and Dewey 2011; Langmead and Salzberg 2012), then filtered out any transcripts that had an Fragments Per Kilobase of transcript per Million mapped reads (FPKM) < 0.5. We then used the Trinity script get_longest_isoform_seq_per_trinity_gene.pl to retain the longest isoform for each transcript. We ran the TransDecoder pipeline included with Trinity to retain only transcripts that had known homology in each transcriptome. We first used TransDecoder.LongOrfs with default parameters. We then conducted blastp searches of the TransDecoder.LongOrfs output against the SWISS-PROT database with an *e*-value cutoff of 1e−5 and hmmscan searches against the Pfam database (31.0) with default parameters. We predicted coding regions with TransDecoder.Predict with parameters –single_best_only –retain_pfam_hits –retain_blastp_hits.

We mapped the trimmed reads to the resulting transcripts with Bowtie2 (v2.3.5.1) (Langmead and Salzberg 2012), marked PCR duplicates with SAMtools markdup (v1.9) (Li *et al.* 2009), and called variants on the transcripts with FreeBayes (v1.3.1) (Garrison and Marth 2012), using all libraries for each species to call variants with parameters -w –standard-filters -C 5 –min-coverage 10. We used vcffilter from vcflib (v1.0.0_rc2) (Garrison 2012) and retained SNPs with a depth of 10, a quality of 20, and at least 2 alternate alleles on both strands. We also filtered out multiallelic SNPs (1.1 and 2.0% of all SNPs in *C. campanidorsalis* and *C. echiniformis*, respectively). We then used ASEReadCounter in GATK (v4.1.9.0) (McKenna *et al.* 2010) to count the number of reads supporting reference and alternate alleles at each SNP for each sample (i.e. for the reads from each individual separately) with parameters –min-depth-of-non-filtered-base 40 –min-base-quality 20 –min-mapping-quality 40. We used RStudio to determine the proportion of homozygous and heterozygous SNPs for each sample, filtering out SNPs in which the proportion of other bases (bases that were not the reference or alternate allele) was larger than 0.05. The vast majority of SNPs were retained after this filtering. We scored any SNPs with an allele bias (proportion of reference to total allele count) between 0.2 and 0.8 as heterozygous and any SNPs with an allele bias less than 0.1 or greater than 0.9 as homozygous. We then compared the frequencies of homozygous and heterozygous SNPs between males and females for each species, with the expectation that if a significant part of the paternally derived genome in males is silenced, then males should show an excess of homozygous expression compared to females (similar to de la Filia *et al*. 2021).

## Results

### Several eriococcid species have lost male somatic heterochromatization

**Fig. 1.**
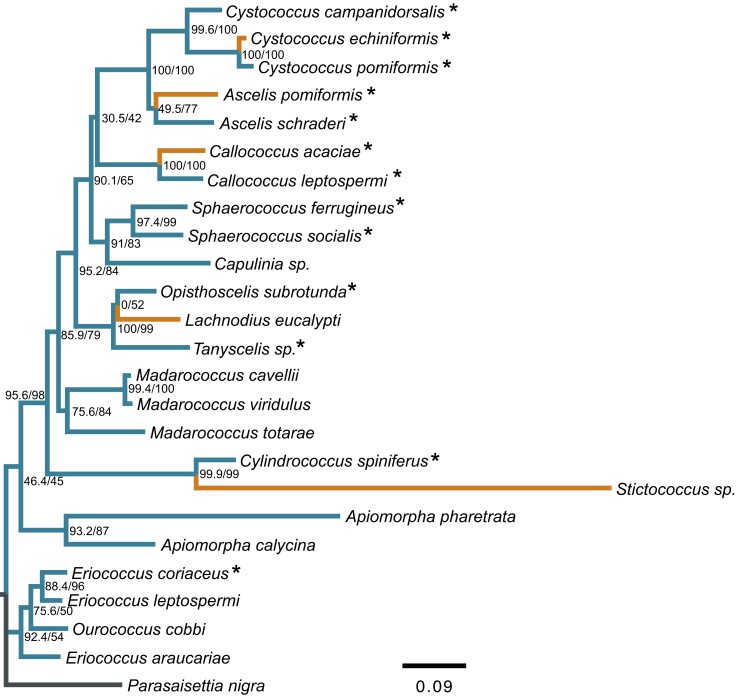
Maximum likelihood phylogeny of eriococcid species for which we have data on whether heterochromatic bodies are present in males. Branches for species without heterochromatic bodies are colored orange (light), while those with heterochromatic bodies are colored blue (dark). The phylogeny was generated with 18S and COI markers and an SYM + I + G4 substitution model with *P. nigra* (Coccidae) included as the outgroup species (branch colored black). Values at nodes show the SH-aLRT/ultrafast bootstrap support with 1,000 replicates. Species examined in this study are indicated with asterisks, whereas the occurrence of heterochromatic bodies for other species was taken from Brown (1967) or Brown (1977).

**Fig. 2.**
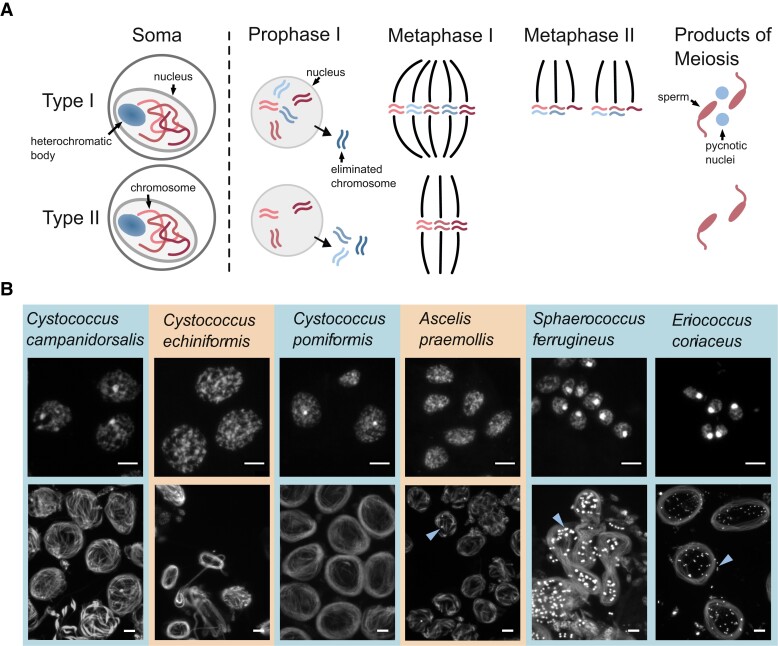
a) Schematic of variation in meiosis in species with Comstockiella PGE. Species with Comstockiella PGE have heterochromatic bodies in somatic cells that contain the paternal chromosomes condensed into a brightly staining ball (blue circle in the periphery of the nucleus). Some (and sometimes all) paternally derived chromosomes are eliminated just prior to meiotic divisions. The number of chromosomes eliminated determines whether there are 1 or 2 divisions in meiosis and whether pycnotic nuclei (containing paternal chromosomes that will not form viable sperm) are present when sperm bundles are forming (type I vs type II). b) DAPI-stained images showing somatic cells (top image) and sperm bundles forming (bottom image) for 6 eriococcid species. There is variation in whether heterochromatic bodies are present in somatic cells (brightly staining circle in nucleus), with species with an orange background lacking heterochromatic bodies (*Cystococcus echiniformis* and *Ascelis praemollis*). There is also variation in whether pycnotic nuclei (indicated with blue arrowheads) are present when sperm bundles are forming. Scale bar = 10 μm.

Of the 13 species we examined, 10 exhibited heterochromatic bodies within somatic tissue of males (Fig. 1). The appearance and size of heterochromatic bodies, however, varied between species and for different nuclei within the same species (Fig. 2; Supplementary Fig. 3). Some species, such as *A. schraderi*, had heterochromatic bodies in all nuclei, while other species, such as *Cystococcus pomiformis*, had a large proportion of cells that lacked heterochromatic bodies (Supplementary Fig. 4). Although it is known that within scale insects with PGE some tissues can lack heterochromatic bodies while others contain them (Nur 1967), we observed substantial variation in the proportion of cells that contain heterochromatic bodies in different species, which should be explored further. In *C. echiniformis*, *Ascelis praemollis*, and *Callococcus acaciae*, we were unable to identify heterochromatic bodies in any males. Species that have lost male somatic heterochromatization, including *L. eucalypti* and *Stictococcus* sp. (Brown 1977), are distributed across the Eriococcidae phylogeny (Fig. 1). This suggests that the loss of somatic paternal chromosome heterochromatization has occurred multiple times independently across the family.

### Male meiosis varies between species in the *Ascelis/Cystococcus* clade

**Fig. 3.**
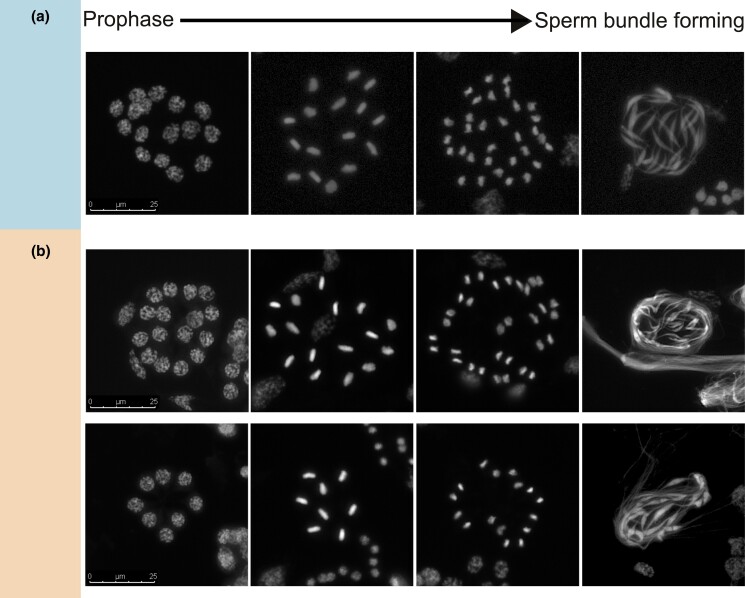
Meiosis in *C. campanidorsalis* a), a species with heterochromatic bodies in somatic cells of males, and *C. echiniformis* b), a close relative without heterochromatic bodies. Images show a sperm cyst containing nuclei throughout different stages of meiosis or following meiosis when sperm is forming. In both species, pycnotic nuclei are absent following meiosis (last image in each panel). In *C. campanidorsalis*, there is 1 division in meiosis where 16 nuclei divide to form 32 sperm a), whereas in *C. echiniformis*, some sperm cysts contain 8 nuclei and some contain 16 nuclei (top vs bottom panel in b)). We also observed some sperm bundles with 32 sperm and some with 16 sperm (top vs bottom panel in b)).

We were able to examine male meiosis in 6 species and found that it varies substantially between eriococcid species. We identified pycnotic nuclei in “*Sphaerococcus*” *ferrugineus* (a.k.a. *Beesonia ferrugineus*) and *E. coriaceus*, indicating that these species exhibit PGE (as noted by Brown (1967) for *E. coriaceus*) (Fig. 2). In *A. praemollis*, which does not have heterochromatic bodies in the soma, sperm bundles occasionally had pycnotic nuclei associated with them, suggesting that this species exhibits PGE despite not having heterochromatic bodies (Fig. 2). However, we could not identify pycnotic nuclei in sperm bundles of any *Cystococcus* species we examined (Figs. 2 and 3), suggesting males might exhibit PGE with only 1 division in meiosis or might not exhibit PGE.

In all *Cystococcus* and *Ascelis* species examined in detail, we made sure to stain a variety of male developmental stages such that we were confident that we were capturing the full meiotic process. Spermatogenesis in scale insects occurs within a cyst where several spermatogonia undergo meiosis concurrently. In *C. campanidorsalis*, we always observed 16 nuclei per sperm cyst at prophase and 32 sperm elongating after meiosis, indicating that *C. campanidorsalis* has 1 division in meiosis and Comstockiella PGE, with the elimination of all paternal chromosomes prior to meiosis (Fig. 3a). *Ascelis praemollis* has 8 nuclei in primary spermatids and 16 sperm forming in each sperm cyst after meiosis, indicating that this species also has Comstockiella PGE and 1 division in meiosis, but sperm cysts contain 8 nuclei in primary spermatids (as noted for *A. schraderi* in Brown (1967)) (Supplementary Fig. 6). In both *C. echiniformis* and *C. pomiformis*, sperm cysts most often had 16 nuclei in prophase sperm cysts, but in the same individuals, we also observed a minority of sperm cysts with 8 nuclei at prophase (Supplementary Figure 5; Supplementary Table 7) (note that in *C. pomiformis*, we were able to view sperm cysts in prophase and sperm bundles forming but not chromosomes dividing). We most often observed 32 sperm following meiosis, but there were a few cases in which we observed 16 sperm following meiosis (Fig. 3b; Supplementary Figs. 4 and 5). This could indicate that in *C. echiniformis* and *C. pomiformis*, the number of nuclei in sperm cysts can vary between 8 and 16. In this case, these species also exhibit PGE since there is only 1 division in meiosis, indicating that paternally inherited chromosomes are eliminated prior to meiosis. Alternatively, *C. echiniformis* and *C. pomiformis* may not exhibit PGE and may undergo 2 divisions in meiosis. We explore these possibilities further in the microsatellite analysis.

### *Cystococcus* species exhibit allele inheritance patterns consistent with PGE

**Fig. 4.**
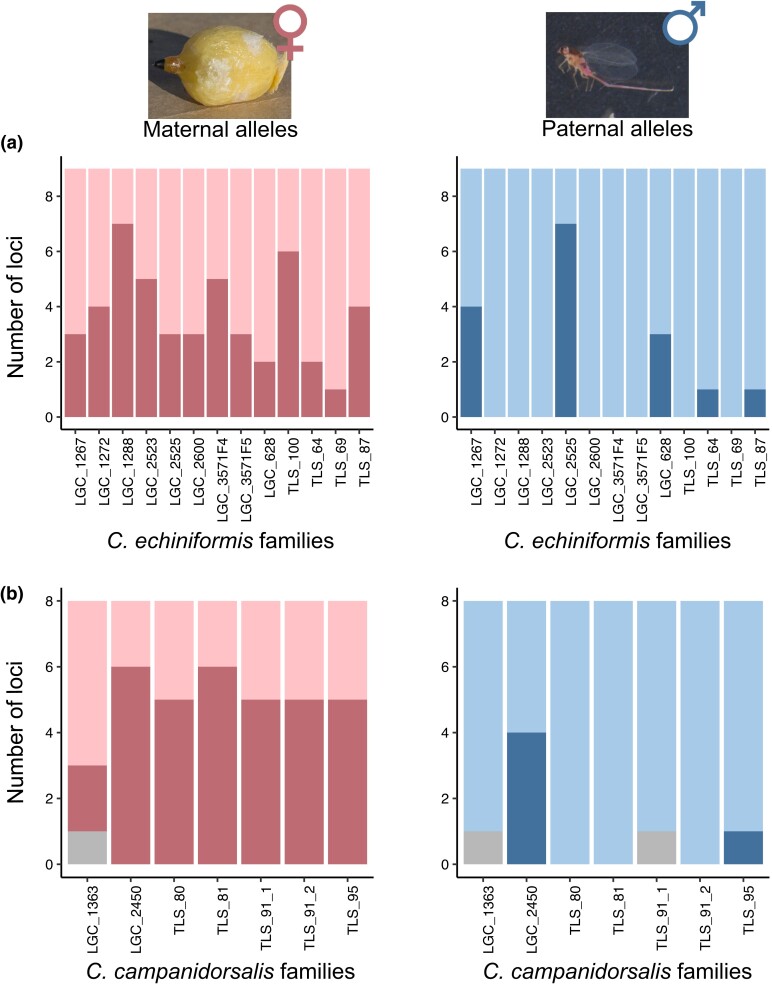
Inheritance patterns for male siblings in broods from a) *C. echiniformis* and b) *C. campanidorsalis*. The inheritance patterns from the mother (pink) and the father (blue) are shown separately. The family ID vs the number of microsatellite markers is shown in each plot, with the darker bar showing the number of loci where broods inherited 2 alleles from their parent and the lighter bar indicating the number of loci where broods inherited 1 allele from their parent (with gray bars indicating the number of loci where the number of alleles received was uncertain due to genotyping errors). For both species, broods were more likely to inherit 2 loci from their mother than their father. An image of a female and male *Cystococcus* is shown at the top of the figure.

To further investigate whether *Cystococcus* species exhibit PGE inheritance, we examined allele inheritance patterns for 9 and 8 microsatellite loci for *C. echiniformis* and *C. campanidorsalis* families, respectively. We analyzed the genotype of broods of male siblings and their mother and inferred the paternal genotype based on the loci found in offspring. We expect all offspring in the brood to inherit the same allele from their mother (i.e. 1 allele collectively) if the mother is homozygous at those loci and each individual to inherit 1 of the 2 maternal alleles (with both alleles found within the brood) if the mother is heterozygous at those loci. If the species exhibits PGE, all offspring will always inherit the same allele from their father (i.e. the brood will inherit 1 allele collectively), while paternal inheritance patterns will be similar to maternal inheritance patterns if the species exhibits Mendelian inheritance. However, as the samples were field-collected, we did not have information about whether females had mated multiply or once, and we therefore inferred this information from the data. On average, *C. echiniformis* broods receive 1.13 alleles from their father and 1.40 alleles from their mother, while *C. campanidorsalis* offspring receive 1.09 alleles from their father and 1.67 alleles from their mother for the loci examined (Fig. 4). The number of cases in which individuals inherited 2 vs 1 allele from their parent differed for alleles inherited from the mother vs the father (glmer: est = 2.38, s.e. = 0.50, *z* = 4.73, *P* < 0.001), but allele inheritance patterns did not differ between *C. campanidorsalis* and *C. echiniformis* (glmer: est = 0.63, s.e. = 0.48, *z* = 1.30, *P* = 0.194) (Supplementary Table 8). This suggests that both species exhibit the same type of inheritance and that both species likely exhibit PGE, as the number of alleles broods inherited from the father was less than the number inherited from the mother.

However, we did observe some instances where a brood inherited 2 paternal alleles for some loci. For the cases where this involved more than 1 microsatellite locus, we investigated whether this was due to multiple mating (i.e. the mother mated with more than 1 male with PGE) or whether this was an indication of a lack of PGE transmission in that family. Under PGE, you would expect that males pass on the same haplotype to all their offspring (i.e. in PGE, you expect linkage disequilibrium to be complete) so that if a female mated with 2 males (e.g. a 2-locus example with 1 male contributing AB and the other ab), her offspring would never inherit Ab or aB. However, a female mating with a single non-PGE male with an aAbB genotype would result in all possible allele combinations (Supplementary Fig. 7). We were able to explore this in 3 families in *C. echiniformis* (families LGC_00628, LGC_01267, and LGC_02525) and 1 family in *C. campanidorsalis* (family LGC_02450). For all 4 of these cases, inheritance of alleles across microsatellite loci was not random (Supplementary Table 9, *P* < 0.01 for all comparisons). For the most part, we only observed 2 of the possible allele combinations in offspring or in a few cases 3 (with the additional combination at low frequency, so likely being the result of a genotyping error) (Supplementary Fig. 7). This indicates that in the cases we were able to examine, broods that had inherited 2 paternal alleles for some loci more likely had mothers that mated multiple times than fathers that lacked PGE. Together, these analyses support our cytogenetic results that both species have PGE transmission.

### *Cystococcus* males exhibit biparental gene expression

**Fig. 5.**
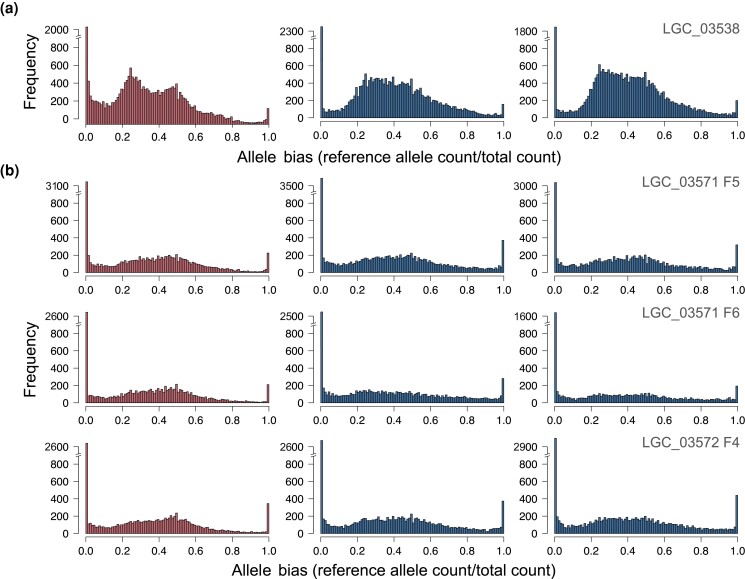
Histograms summarizing allele expression for mothers (red/ first image of each row) and 2 sons (blue) for each family of *C. campanidorsalis* a) and *C. echiniformis* b). The frequency of the allele bias (count of the called reference allele/total allele count) for biallelic SNPs is shown, with alleles with homozygous (haploid) expression shown at either 0 or 1 and alleles with heterozygous expression shown from 0.2 to 0.8. In males with heterochromatic bodies, we would expect primarily homozygous (haploid) allele expression. Instead, mothers and sons have similar expression patterns in both species. The family ID is shown in the top right corner of each panel.

We also examined whether the loss of heterochromatic bodies from somatic cells of males indicates that males exhibit biparental gene expression rather than predominantly maternal expression of genes, which we expect for males with heterochromatic bodies (de la Filia *et al.* 2021). To do so, we compared patterns of heterozygosity between females (mothers) and males (sons) of 1 *C. campanidorsalis* family and 3 *C. echiniformis* families, where we would expect an excess of homozygous SNPs in males if their paternal genome was either completely or partially silenced. We identified 39,439 biallelic SNPs in *C. campanidorsalis* and 30,184 biallelic SNPs in *C. echiniformis.* We filtered reads from each sample that did not meet quality filters and excluded SNP positions from the final analysis that did not have sufficient depth of quality reads, resulting in slightly different numbers of SNPs that we considered for each individual (see Supplementary Table 10). *Cystococcus echiniformis* mothers exhibited heterozygous expression (20–80% expression of the nonreference allele) for 62.2–63.5% of SNPs, while sons exhibited heterozygous expression for 59.0–66.3% of SNPs (Fig. 5b; Supplementary Table 10). For *C. campanidorsalis*, the mother exhibited heterozygous expression for 77.0% of SNPs, while the 2 sons exhibited heterozygous expression for 85.4 and 85.5% of SNPs (Fig. 5a). Overall, we found that sons exhibited biparental (heterozygous) allele expression patterns for a substantial number of SNPs in both *C. echiniformis* and *C. campanidorsalis*, suggesting that *C. campanidorsalis* males express alleles inherited from their father despite having heterochromatic bodies in somatic tissue.

## Discussion

Asymmetric chromosome inheritance has evolved in numerous lineages (Ross *et al*. 2022). PGE and haplodiploidy are 2 common asymmetric systems, both involving exclusive transmission of maternally derived chromosomes in males. How such a system evolves, and whether reversions back to more typical Mendelian systems can occur, is currently unclear. For PGE in scale insects, conflict between the sexes over the transmission of the parental genomes through male offspring is thought to have led to its initial evolution, where the maternal genome displays meiotic drive (Brown 1964; Haig 1993). However, ongoing intragenomic conflict between the maternal and paternal genomes within males is thought to have driven the transcriptional silencing of the paternal genome through heterochromatization or the earlier elimination of paternal chromosomes (Herrick and Seger 1999; Ross *et al.* 2010). In males that have retained chromosomes inherited from their father, there should be strong ongoing selection for paternally inherited chromosomes to regain transmission, which could have a number of consequences. First, it could drive the loss and/or silencing of the paternal genome through counter adaptations from the maternally inherited chromosomes. On the other hand, it may eventually lead to transmission of some or all of the paternally inherited chromosomes through sperm. However, given the significant mechanistic changes associated with the evolution of PGE (i.e. meiosis, spermatogenesis, ploidy, and sex determination), transitions back to Mendelian inheritance (i.e. paternally inherited chromosomes regaining transmission through males) may be difficult or even impossible. We explored whether there is evidence for transmission of paternally inherited chromosomes through sperm (i.e. loss of PGE) or paternal chromosome expression in males using cytogenetic, genotyping, and gene expression analyses in eriococcid scale insects: the only lineage in which PGE is thought to have been lost in several species.

Since eriococcid species ancestrally exhibit Comstockiella PGE, in which paternally inherited chromosomes are heterochromatized in males, 1 way of identifying changes in the presence/mechanism of PGE is by staining male somatic tissue to determine if heterochromatic bodies are present. We analyzed samples from 13 species and found that in addition to the 2 species that were previously found to have lost heterochromatic bodies (*L. eucalypti* and *Stictococcus* sp.), at least 3 other species within this family have also lost paternal somatic heterochromatization. Intriguingly, we also found evidence that the size of heterochromatic bodies, relative to the rest of the nuclei, differs both within and between species in this family (Fig. 2; Supplementary Fig. 3). We currently do not understand why this variation may exist, but it may hint at ongoing conflict between maternal and paternal chromosomes in this family. As heterochromatic bodies contain all paternally inherited chromosomes, condensed into a heterochromatic “ball,” loss or variation in the size of the heterochromatic bodies might indicate that some or all paternally inherited chromosomes may be escaping transcriptional suppression in males and therefore may have a greater influence over transmission/reproduction.

We found, through staining male reproductive tissue undergoing meiosis, that a lack of heterochromatic bodies does not necessarily mean a lack of PGE. Two species that lacked heterochromatic bodies, *C. echiniformis* and *A. praemollis*, have only 1 division in meiosis, suggesting that paternally inherited chromosomes are not passed on but instead lost prior to meiosis (Fig. 3; Supplementary Fig. 6). Additionally, our microsatellite inheritance data showed that *C. echiniformis* exhibits inheritance patterns consistent with PGE. Overall, this indicates that these species have transitioned to a different type of PGE, not previously reported in scale insects, but have not lost this type of reproduction entirely. This result opens debate over whether *L. eucalypti* and *Stictococcus* sp. really lack PGE or whether earlier reports of meiosis in these species were misinterpreted. The evidence that *L. eucalypti* and *Stictococcus* sp. lacked PGE transmission was based on cytological data only (and with just a few available specimens) (Brown 1977). It is therefore possible that these species exhibit a similar type of PGE to *C. echiniformis*, where variation in the number of nuclei per sperm cyst could be easily mistaken for evidence of 2 rather than 1 division in meiosis. Further investigation of chromosome transmission in *L. eucalypti* and *Stictococcus* sp. is needed to corroborate earlier conclusions that PGE was lost. Unfortunately, this was not possible in our study: *L. eucalypti* is scarce, the only specimen we were able to find was an immature female, and the *Stictococcus* sp. studied previously was undescribed, so it was not possible to follow up this work.

Herrick and Seger (1999) suggested that the elimination of paternal chromosomes just prior to meiosis evolved to prevent paternal chromosomes from resisting maternal chromosome drive *during* meiosis. Therefore, variation in the characteristics of PGE may indicate ongoing conflict between the maternal and paternal halves of the genome in males. Our staining results indicate that in *C. echiniformis* and *A. praemollis*, the majority of paternal chromosomes are likely eliminated entirely before meiosis, as otherwise we would expect to see 2 divisions in meiosis rather than 1 (Fig. 3; Supplementary Figs. 6 and 8). Although this may indicate a lack of opportunity for paternally inherited chromosomes to be transmitted to future generations, it is not conclusive that paternally transmitted chromosomes are never transmitted to offspring. In Comstockiella PGE systems, if most but not all of the paternal chromosomes are eliminated just prior to meiosis, there is 1 division in meiosis, with the remaining paternal chromosomes presumed to be eliminated afterward (Nur 1980), and in mealybugs, which have lecanoid PGE, occasional paternal chromosome leakage does occur through sperm (de la Filia *et al.* 2019). Therefore, there may be occasional leakage of paternally inherited chromosomes through sperm in eriococcid species. Although it would be fascinating to see if this ever happens, it would require a larger-scale inheritance analysis than we undertook in this study, with larger sample sizes and more markers than we were able to use. It will also be challenging because, unlike mealybugs that can be easily cultured under laboratory condition, the species considered in this study feed exclusively on trees of the family Myrtaceae and have not been successfully reared in the lab despite several attempts.

We also found that males of both species exhibit substantial biparental gene expression. This result is surprising, as *C. campanidorsalis* has heterochromatic bodies, which are associated with substantial (although not complete) transcriptional suppression of paternally inherited chromosomes in males in the mealybug *Planococcus citri* (de la Filia *et al.* 2021). Variation in the presence and size of heterochromatic bodies may be related to the expression levels of paternally derived chromosomes in males. The relative size of the heterochromatic bodies that we observed in all *Cystococcus* species was smaller than those observed in the mealybug *P. citri*, which could indicate that not all paternal chromosomes are part of the heterochromatic body. Alternately, the way that we assessed species for the presence of heterochromatic bodies, by scoring any species in which we observed heterochromatic bodies as possessing this trait, makes sense when scoring for the presence of PGE but perhaps not when thinking about expression of paternally inherited chromosomes. For instance, in *C. pomiformis*, a close relative to *C. campanidorsalis*, we noted that some cells have heterochromatic bodies and some cells do not (Supplementary Fig. 4). Therefore, perhaps not all tissues exhibit heterochromatic bodies in *C. campanidorsalis*, resulting in a significant amount of biparental expression at the whole body level. Tissue-specific heterochromatization has been previously noted in other scale insect species, including *P*. *citri*, where some somatic and germline tissues do not possess heterochromatic bodies (although it is not clear how this relates to expression in those tissues) (Nur 1967; de la Filia *et al.* 2021).

The fact that males in *C. echiniformis* and *C. campanidorsalis* both express paternal chromosomes in somatic cells is intriguing. Eriococcid species evolved from an ancestor with somatic heterochromatization (Nur 1980; Ross *et al.* 2010), suggesting that the ancestor likely showed limited expression of paternal alleles, like mealybugs (de la Filia *et al.* 2021). Therefore, re-evolving biparental expression from a uniparental chromosome expression system may be possible in PGE species and leads to a transition from haploid to diploid male gene expression. Although the mechanism of sex determination in scale insects is unclear (as no species in this clade have heteromorphic sex chromosomes), somatic heterochromatization was suggested to be an important aspect of the sex determination system (Buglia *et al.* 2009). Our results indicate that this may not be the case. However, our study was designed to assess broad patterns of expression, specifically whether males exhibited haploid or diploid chromosome expression on a whole-genome scale. Because of this, there are still many questions to be answered about chromosome expression in *Cystococcus* species. For instance, are there any chromosomes/genes in which expression is strictly maternal and do all tissues exhibit the same expression patterns? How has the shift to biparental chromosome expression affected dosage compensation (i.e. overall gene expression levels), and do any related species have strictly maternal chromosome expression? Perhaps a good species to explore this last question in is *A. schraderi*, which is closely related to *Cystococcus* species (Semple *et al.* 2015) and exhibits large heterochromatic bodies in most tissues (Fig. 1; Supplementary Fig. 3).

The type of PGE found in *C. echiniformis*, with no heterochromatic bodies in somatic cells, was previously only known from head and body lice (de la Filia *et al.* 2018). Our results suggest that the evolution of PGE may have followed a similar trajectory in lice and scale insects. PGE has been characterized in few lice species (only head/body lice and *Liposcelis* booklice) (Hodson *et al.* 2017; de la Filia *et al.* 2018). However, all parasitic lice exhibit a peculiar type of meiosis in males (reviewed in White (1973)), and as head and body lice are nested within the monophyletic clade of parasitic lice to which booklice form the outgroup (Yoshizawa and Johnson 2010), the entire clade likely exhibits PGE. Booklice have a similar type of PGE to many scale insects (which is thought to be the ancestral form of PGE in this clade), with heterochromatic bodies in somatic cells of males (Hodson *et al.* 2017). The evolutionary trajectory seems to be similar in lice and scale insects, with some species exhibiting modified PGE systems with a loss of heterochromatic bodies and biparental gene expression in males, nested within clades where species exhibit more typical PGE systems with heterochromatic bodies and suppressed paternal gene expression. More in-depth examination of the mechanism of PGE in both clades can provide insight into general trends in the evolution of PGE and asymmetric inheritance.

### Concluding remarks

Our results indicate that the mechanism of PGE is variable in scale insects, but there is no evidence for transitions from PGE to Mendelian inheritance. Also, the 2 scale insect species in which PGE was previously thought to be lost should be re-examined. Understanding more about the mechanism of reproduction in species with PGE helps us determine how likely it would be for PGE to transition back to Mendelian inheritance. For instance, in scale insects, we know that the transition to PGE must have involved a shift in how sex determination occurs in this lineage, as the ancestral XO sex determination system would result in female-only offspring with a shift to PGE transmission dynamics in the absence of a shift in the sex determination system. However, we still do not understand how sex determination occurs in scale insects and, therefore, whether it would be difficult to lose PGE transmission dynamics and still retain viable individuals that are able to reproduce in this lineage. This is also true in other lineages with PGE. Therefore, surveys of the characteristics of PGE and reproduction in taxonomically diverse species with asymmetric inheritance provide a valuable foundation from which to answer questions about the evolution of non-Mendelian inheritance.

Although we did not find evidence for a loss of PGE in eriococcid scale insects, we did find evidence for a previously unknown mechanism of PGE within scale insects. In *C. echiniformis*, males exhibited PGE transmission dynamics despite a lack of heterochromatic bodies, and in both *C. echiniformis* and *C. campanidorsalis*, we find that paternally inherited chromosomes are expressed in males, which was not previously thought to occur. Future work into whether the shift from haploid to diploid chromosome expression in *Cystococcus* species is complete, and how expression patterns compare to species with haploid expression, is needed. Eriococcidae are therefore an ideal clade of insects for studying how variability in epigenetic chromosome modification affects expression, and changes in ploidy and associated dosage compensation mechanisms evolve.

## Supplementary Material

iyad090_Supplementary_Data

## Data Availability

Scripts associated with this study are deposited on GitHub (https://github.com/chodson/eriococcidae_PGE) and archived on Zenodo (doi:10.5281/zenodo.7682169). Sequences used in the phylogenetic analysis and RNA-seq reads and transcriptome assemblies are deposited at European Nucleotide Archive (ENA) under the project accession: PRJEB60048. Supplemental material available at GENETICS online.

## References

[iyad090-B1] Bachtrog D, Mank JE, Peichel CL, Kirkpatrick M, Otto SP, Ashman TL, Hahn MW, Kitano J, Mayrose I, Ming R , et al Sex determination: why so many ways of doing it? PLoS Biol. 2014;12(7): e1001899 7). doi:10.1371/journal.pbio.1001899 PMC4077654 24983465

[iyad090-B2] Bates D, Mächler M, Bolker B, Walker S. Fitting linear mixed-effects models using lme4. J Stat Softw. 2015;67:1–48.

[iyad090-B3] Blackmon H, Ross L, Bachtrog D. Sex determination, sex chromosomes, and karyotype evolution in insects. J Hered. 2017;108(4):78–93. doi:10.1093/jhered/esw047 27543823 PMC6281344

[iyad090-B4] Bongiorni S, Fiorenzo P, Pippoletti D, Prantera G. Inverted meiosis and meiotic drive in mealybugs. Chromosoma. 2004;112(7):331–341. doi:10.1007/s00412-004-0278-4 15095094

[iyad090-B5] Bongiorni S, Mazzuoli M, Masci S, Prantera G. Facultative heterochromatization in parahaploid male mealybugs: involvement of a heterochromatin-associated protein. Development. 2001;128(19):3809–3817. doi:10.1242/dev.128.19.3809 11585806

[iyad090-B6] Brown SW . Automatic frequency response in the evolution of male haploidy and other coccid chromosome systems. Genetics. 1964;49(5):797–817. doi:10.1093/genetics/49.5.797 17248212 PMC1210615

[iyad090-B7] Brown SW . Chromosome systems of the Eriococcidae (Coccoidea-Homoptera): I. A survey of several genera. Chromosoma. 1967;22(2):126–150. doi:10.1007/BF00326725

[iyad090-B8] Brown SW . Adaptive status and genetic regulation in major evolutionary changes of coccid chromosome systems. Nucleus. 1977;20:145–157.

[iyad090-B9] Brown SW, Bennett FD. On sex determination in the diaspine scale *Pseudaulacaspis pentagona* (Targ. Coccoidea). Genetics. 1957;42(4):510–523. doi:10.1093/genetics/42.4.510 17247712 PMC1209846

[iyad090-B10] Brown SW, Nelson-Rees WA. Radiation analysis of a lecanoid genetic system. Genetics. 1961;46(8):983–1007. doi:10.1093/genetics/46.8.983 17248058 PMC1210258

[iyad090-B11] Buglia GL, Dionisi D, Ferraro M. The amount of heterochromatic proteins in the egg is correlated with sex determination in *Planococcus citri* (Homoptera. Coccoidea). Chromosoma. 2009;118(6):737–746. doi:10.1007/s00412-009-0231-7 19636581

[iyad090-B12] Bull JJ . An advantage for the evolution of male haploidy and systems with similar genetic transmission. Heredity (Edinb). 1979;43(3):361–381. doi:10.1038/hdy.1979.88

[iyad090-B13] Bull JJ . Evolution of Sex Determination Mechanisms. Menlo Park: Benjamin/Cummings Publishing Company; 1983.

[iyad090-B14] Burt A, Trivers R. Genes in Conflict: The Biology of Selfish Genetic Elements. Cambridge: Belknap Press of Harvard University Press; 2006.

[iyad090-B15] Chen S, Zhou Y, Chen Y, Gu J. fastp: an ultra-fast all-in-one FASTQ preprocessor. Bioinformatics. 2018;34(17):i884–i890. doi:10.1093/bioinformatics/bty560 30423086 PMC6129281

[iyad090-B16] Cook LG . Extraordinary and extensive karyotypic variation: a 48-fold range in chromosome number in the gall-inducing scale insect *Apiomorpha* (Hemiptera: Coccoidea: Eriococcidae). Genome. 2000;43(2):255–263. doi:10.1139/gen-43-2-255 10791813

[iyad090-B17] Cook LG, Gullan PJ. The gall-inducing habit has evolved multiple times among the eriococcid scale insects (Sternorrhyncha: Coccoidea: Eriococcidae). Biol J Linn Soc. 2004;83:441–452. doi:10.1111/j.1095-8312.2004.00396.x.

[iyad090-B18] Cook LG, Gullan PJ, Trueman HE. A preliminary phylogeny of the scale insects (Hemiptera: Sternorrhyncha: Coccoidea) based on nuclear small-subunit ribosomal DNA. Mol Phylogenet Evol. 2002;25(1):43–52. doi:10.1016/S1055-7903(02)00248-8 12383749

[iyad090-B19] de la Filia AG, Andrewes S, Clark JM, Ross L. The unusual reproductive system of head and body lice (*Pediculus humanus*). Med Vet Entomol. 2018;32(2):226–234. doi:10.1111/mve.12287 29266297 PMC5947629

[iyad090-B20] de la Filia AG, Bain SA, Ross L. Haplodiploidy and the reproductive ecology of arthropods. Curr Opin Insect Sci. 2015;9:36–43. doi:10.1016/j.cois.2015.04.018. 32846706

[iyad090-B21] de la Filia AG, Fenn-Moltu G, Ross L. No evidence for an intragenomic arms race under paternal genome elimination in *Planococcus* mealybugs. J Evol Biol. 2019;32(5):491–504. doi:10.1111/jeb.13431 30776169

[iyad090-B22] de la Filia AG, Mongue AJ, Dorrens J, Lemon H, Laetsch DR, Ross L. Males that silence their father's genes: genomic imprinting of a complete haploid genome. Mol Biol Evol. 2021;38(6):2566–2581. doi:10.1093/molbev/msab052 33706381 PMC8136510

[iyad090-B23] Du Bois AM . Chromosome behavior during cleavage in the eggs of *Sciara coprophila* (Diptera) in the relation to the problem of sex determination. Z Für Zellforsch Mikrosk Anat. 1933;19(3):595–614. doi:10.1007/BF00393361

[iyad090-B24] Folmer O, Black M, Hoeh W, Lutz R, Vrijenhoek R. DNA primers for amplification of mitochondrial cytochrome c oxidase subunit I from diverse metazoan invertebrates. Mol Mar Biol Biotechnol 1994;3(5):294–299. 7881515

[iyad090-B25] Gardner A, Ross L. Mating ecology explains patterns of genome elimination. Ecol Lett. 2014;17(12):1602–1612. doi:10.1111/ele.12383 25328085 PMC4240462

[iyad090-B26] Garrison E. 2012. vcflib: a C++ library for parsing and manipulating VCF files. GitHub. https://github.com/ekg/vcflib.

[iyad090-B27] Garrison E, Marth G. 2012. Haplotype-based variant detection from short-read sequencing. ArXiv12073907 Q-Bio.

[iyad090-B28] Gavrilov IA . A catalog of chromosome numbers and genetic systems of scale insects (Homoptera: Coccinea) of the world. Isreal J Entomol. 2007;37(2):1–45.

[iyad090-B29] Grabherr MG, Haas BJ, Yassour M, Levin JZ, Thompson DA, Amit I, Adiconis X, Fan L, Raychowdhury R, Zeng Q. Trinity: reconstructing a full-length transcriptome assembly from RNA-Seq data without a reference genome. Nat Biotechnol. 2011;29(3):644–652. doi:10.1038/nbt.1883 21572440 PMC3571712

[iyad090-B30] Guindon S, Dufayard JF, Lefort V, Anisimova M, Hordijk W, Gascuel O. New algorithms and methods to estimate maximum-likelihood phylogenies: assessing the performance of PhyML 3.0. Syst Biol. 2010;59(3):307–321. doi:10.1093/sysbio/syq010 20525638

[iyad090-B31] Gullan PJ, Cockburn A. Sexual dichronism and intersexual phoresy in gall-forming coccoids. Oecologia. 1986;68:632–634. doi:10.1007/BF00378784. 28311725

[iyad090-B32] Gullan PJ, Cook LG. Phylogeny and higher classification of the scale insects (Hemiptera: Sternorrhyncha: Coccoidea). Zootaxa. 2007;1668(1):413–425. doi:10.11646/zootaxa.1668.1.22

[iyad090-B33] Haig D . The evolution of unusual chromosomal systems in coccoids: extraordinary sex ratios revisited. J Evol Biol. 1993;6(1):69–77. doi:10.1046/j.1420-9101.1993.6010069.x

[iyad090-B34] Hartl DL, Brown SW. The origin of male haploid genetic systems and their expected sex ratio. Theor Popul Biol. 1970;1(2):165–190. doi:10.1016/0040-5809(70)90033-X 5538189

[iyad090-B35] Herrick G, Seger J. Genomic Imprinting: An Interdisciplinary Approach. In Imprinting and paternal genome elimination in insects. Berlin: Springer; 1999. p. 41–71. 10.1007/978-3-540-69111-2_3 10339741

[iyad090-B36] Hoang DT, Chernomor O, von Haeseler A, Minh BQ, Vinh LS. UFBoot2: improving the ultrafast bootstrap approximation. Mol Biol Evol. 2018;35(2):518–522. doi:10.1093/molbev/msx281 29077904 PMC5850222

[iyad090-B37] Hodson CN, Hamilton PT, Dilworth D, Nelson CJ, Curtis CI, Perlman SJ. Paternal genome elimination in *Liposcelis* booklice (Insecta: Psocodea). Genetics. 2017;206(2):1091–1100. doi:10.1534/genetics.117.199786 28292917 PMC5499165

[iyad090-B38] Kalyaanamoorthy S, Minh BQ, Wong TKF, von Haeseler A, Jermiin LS. ModelFinder: fast model selection for accurate phylogenetic estimates. Nat Methods. 2017;14(6):587–589. doi:10.1038/nmeth.4285 28481363 PMC5453245

[iyad090-B39] Katoh K, Standley DM. MAFFT multiple sequence alignment software version 7: improvements in performance and usability. Mol Biol Evol. 2013;30(4):772–780. doi:10.1093/molbev/mst010 23329690 PMC3603318

[iyad090-B40] Kubai DF . Meiosis in *Sciara coprophila*: structure of the spindle and chromosome behavior during the first meiotic division. J Cell Biol. 1982;93(3):655–669. doi:10.1083/jcb.93.3.655 7118997 PMC2112171

[iyad090-B41] Langmead B, Salzberg SL. Fast gapped-read alignment with Bowtie 2. Nat Methods. 2012;9(4):357–359. doi:10.1038/nmeth.1923 22388286 PMC3322381

[iyad090-B42] Li B, Dewey CN. RSEM: accurate transcript quantification from RNA-Seq data with or without a reference genome. BMC Bioinformatics. 2011;12:323. 10.1186/1471-2105-12-323 21816040 PMC3163565

[iyad090-B43] Li H, Handsaker B, Wysoker A, Fennell T, Ruan J, Homer N, Marth G, Abecasis G, Durbin R, 1000 Genome Project Data Processing Subgroup etal The sequence alignment/map format and SAMtools. Bioinformatics 2009;25(16):2078–2079. doi:10.1093/bioinformatics/btp352 19505943 PMC2723002

[iyad090-B44] McKenna A, Hanna M, Banks E, Sivachenko A, Cibulskis K, Kernytsky A, Garimella K, Altshuler D, Gabriel S, Daly M. The Genome Analysis Toolkit: a MapReduce framework for analyzing next-generation DNA sequencing data. Genome Res. 2010;20(9):1297–1303. doi:10.1101/gr.107524.110 20644199 PMC2928508

[iyad090-B45] Meglécz E et al QDD version 3.1: a user-friendly computer program for microsatellite selection and primer design revisited: experimental validation of variables determining genotyping success rate. Mol Ecol Resour. 2014;14(6):1302–1313. doi:10.1111/1755-0998.12271 24785154

[iyad090-B46] Minh BQ, Schmidt HA, Chernomor O, Schrempf D, Woodhams MD, von Haeseler A, Lanfear R. IQ-TREE 2: new models and efficient methods for phylogenetic inference in the genomic era. Mol Biol Evol. 2020;37(5):1530–1534. doi:0.1093/molbev/msaa015 32011700 10.1093/molbev/msaa015 PMC7182206

[iyad090-B47] Moghadam HK, Harrison PW, Zachar G, Székely T, Mank JE. The plover neurotranscriptome assembly: transcriptomic analysis in an ecological model species without a reference genome. Mol Ecol Resour. 2013;13(4):696–705. doi:10.1111/1755-0998.12096 23551815

[iyad090-B48] Normark BB . The evolution of alternative genetic systems in insects. Annu Rev Entomol. 2003;48(1):397–423. doi:10.1146/annurev.ento.48.091801.112703 12221039

[iyad090-B49] Nur U . Reversal of heterochromatization and the activity of the paternal chromosome set in the male mealybug. Genetics. 1967;56(3):375–389. doi:10.1093/genetics/56.3.375 6052566 PMC1211625

[iyad090-B50] Nur U . Heterochromatization and euchromatization of whole genomes in scale insects (Coccoidea: Homoptera). Dev Suppl. 1990;Supplement:29–34. doi:10.1242/dev.108.Supplement.29 2090427

[iyad090-B51] Nur U . Insect Cytogenetics Blackwell Oxford. p. 97–117. Evolution of unusual chromosome systems in scale insects (Coccoidea: Homoptera); 1980.

[iyad090-B52] Park DS, Suh SJ, Oh HW, Hebert PD. Recovery of the mitochondrial COI barcode region in diverse Hexapoda through tRNA-based primers. BMC Genomics. 2010;11(1):423. doi:10.1186/1471-2164-11-423 20615258 PMC2996951

[iyad090-B449] R Core Team. R: A Language and environment for statistical computing. R Foundation for Statistical Computing, Vienna, Austria; 2020.

[iyad090-B53] Ross L, Davies NG, Gardner A. How to make a haploid male. Evol Lett. 2019;3(2):173–184. doi:10.1002/evl3.107 31289691 PMC6591549

[iyad090-B54] Ross L, Mongue AJ, Hodson CN, Schwander T. Asymmetric inheritance: the diversity and evolution of non-Mendelian reproductive strategies. Annu Rev Ecol Evol Syst. 2022;53:1–23. doi:10.1146/annurev-ecolsys-021822-010659

[iyad090-B55] Ross L, Pen I, Shuker DM. Genomic conflict in scale insects: the causes and consequences of bizarre genetic systems. Biol Rev. 2010;85(4):807–828. doi:10.1111/j.1469-185X.2010.00127.x 20233171

[iyad090-B56] Semple TL, Gullan PJ, Hodgson CJ, Hardy NB, Cook LG. Systematic review of the Australian ‘bush-coconut’ genus *Cystococcus* (Hemiptera: Eriococcidae) uncovers a new species from Queensland. Invertebr Syst. 2015;29(3):287. doi:10.1071/IS14061

[iyad090-B57] von Dohlen CD, Moran NA. Molecular phylogeny of the Homoptera: a paraphyletic taxon. J Mol Evol. 1995;41(2):211–223. doi:10.1007/BF00170675 7666451

[iyad090-B58] Waterhouse RM et al BUSCO applications from quality assessments to gene prediction and phylogenomics. Mol Biol Evol. 2018;35(3):543–548. doi:10.1093/molbev/msx319 29220515 PMC5850278

[iyad090-B59] White M . Animal Cytology and Evolution. Cambridge: Cambridge University Press; 1973.

[iyad090-B60] Yoshizawa K, Johnson KP. How stable is the “Polyphyly of Lice” hypothesis (Insecta: Psocodea)?: a comparison of phylogenetic signal in multiple genes. Mol Phylogenet Evol. 2010;55(3):939–951. doi:10.1016/j.ympev.2010.02.026 20211746

