# Supplementary material for: Are asymmetric inheritance systems an evolutionary trap? Transitions in the mechanism of paternal genome loss in the scale insect family Eriococcidae
Source: Genetics. 2023 May 15;224(3):iyad090. doi: 10.1093/genetics/iyad090 (PMC10324942; doi:10.1093/genetics/iyad090)
Supplement: iyad090_Supplementary_Data [file iyad090_supplementary_data.pdf]

Supplementary Information for:

Are asymmetric inheritance systems an evolutionary trap? Transitions in the mechanism of genome loss in the scale insect family Eriococcidae

Christina N Hodson, Alicia Toon, Lyn Cook, Laura Ross

Supplementary Methods

Microsatellite primer design

DNA was extracted from a portion of the body wall of an adult female using a CTAB DNA extraction protocol. For the DNA extraction, we first added 600µl of CTAB (Sigma-Aldrich) and 5µl of proteinase K, and incubated the sample overnight at 56°C. We then centrifuged the sample at 13,000rpm for 3 minutes and transferred the supernatant to a new vial. We then added 500 µL of chloroform, inverted the tube 10 times, then centrifuged the sample for 3 min (at 13,000 rpm) and transferred the supernatant to a new tube. We repeated the chloroform step a second time, then added 200µl of a binding buffer (573.18g GuHCL, 500ml NH4Ac pH6, 500ml H_2O), along with 200µl of 100% EtOH, after which I vortexed the mixture and transferred it into a spin column. Then we followed the manufacturer's instructions from the Isolate II genomic DNA kit (Bioline) from the first ethanol wash step, finally adding 50µl of EB buffer to elute the DNA.

For *C. campanidorsalis,* TruSeq DNA library preparation and Illumina 150 base-pair paired-end sequencing (HiSeq2500) was completed at Macrogen Inc. (Republic of Korea) using one sixth of a lane. Potential SSR loci were identified using the QDD2 pipeline software package (Meglécz *et al.*, 2009) and BLAST to ensure that none were within known coding regions. Using Primer3 (Untergasser *et al.*, 2012) within QDD2, we designed primer pairs to amplify fragments between 90 and 400 base pairs in length, with a melting temperature between 57 °C and 63 °C and defaults for other parameters.

For *C. echiniformis*, we sequenced the sample at Edinburgh Genomics with MiSeq (250bp paired end seq with 350bp inserts) to generate low-coverage whole genome sequence data. We trimmed reads with fastp with parameters --cut_by_quality5 --

cut_by_quality3 --cut_window_size 4 --cut_mean_quality 20 (v 0.12.3) (Chen *et al.*, 2018), and assembled the reads using default settings with CLC assembly cell (v5.0.0, Qiagen). We used this assembly to generate primers with QDD (Meglecz *et al.*, 2014). For primer generation, we used the default setting with the exception that we set the minimum PCR product size to 120bp. We also did not do the optional contamination check step (step4 in Meglecz *et al.* 2014). Instead, we chose simple trinucleotide microsatellites as the target regions, generated primer sets that produced amplicon sizes ranging from 120-300bp, and blasted the contigs these primers were found on to the nr nucleotide database on NCBI, excluding any primers from contigs that blasted to non-Metazoan species. For both species, we chose 24 primer pairs and used the nine that produced the most consistent signals when multiplexed in groups of three primer pairs per PCR reaction.

Microsatellite PCR and thermocycling conditions

For PCR reactions, we used the Type-it microsatellite PCR kit (Qiagen), following the manufacturers guideline with a few variations due to the use of M13 fluorescent primers (Schuelke, 2000). We conducted PCR reactions in a total of 15μl, with 7.5 μl Type-it Mastermix, 0.375μl of the forward primer mix (with each primer at a concentration of 2μM), 1.5μl of the reverse primer mix (also at 2μM), 0.5μl of the M13 fluorescent primer (6FAM or VIC, at 5μM), 3.625μl, and 1.5μl of DNA. The thermocycling conditions for the microsatellite PCR was as follows (following guidelines from Schuelke, 2000): 94°C x 5 min, 30 x (94°C x 30sec, 56°C x 45sec, 72°C x 45sec), 8 x (94°C x 30sec, 53°C x 45sec, 72°C x 45sec), 72°C x 10min.

We aimed to genotype 20 males and the mother of each family for each microsatellite loci. However, due to differences in the number of sons collected for each family, and some DNA extractions or primer sets not working on some males, we analysed fewer than 20 males for some families (or for some primers for a family). For family TLS_091, we analysed 13 males, for family TLS_087 we analysed 15 males, for families LGC_01363 and LGC_02525 we analysed 17 males, for family TLS_095 we analysed 18 males, and for family TLS_100 we analysed 19 males.

RNA extractions for gene expression analysis

For RNA extractions from females, we extracted RNA from a small amount of body tissue from the females as we did not want contamination from germ tissue in the sample. We first took the female tissue out of RNAlater and rinsed it briefly in sterile 1X PBS before

adding 50μl of Trizol to the sample and crushing the sample with a micropestle. We then added 950μl of Trizol and 200μl of chloroform, shook the sample by hand, transferred the supernatant to a new tube, added another 200μl of chloroform, and transferred the supernatant into a new tube for a second time. We then added 500ul of isopropanol and 1μl of linear acrylamide and stored the sample overnight at -20°C. The next day, we centrifuged the sample at 4°C for 15 min, removed the isopropanol from the samples, and added 1ml of freshly prepared 70% EtOH. We inverted the sample several times, centrifuged at 4°C for 15 min (13,000 rpm), and removed the EtOH with a pipette. We did the EtOH cleaning step twice for each sample. We dried the pellet at room temperature, then resuspended the pellet in 40ul distilled H_2O. We then performed a gDNA digestion, adding 1μl 10X reaction buffer with $MgCl_2$ (ThermoScientific), 1μl DNase I, 8μl water, and 0.25μl of RNase inhibitor for every 1μl of RNA in the sample. We heated the sample at 37°C for 30 min, then added 1μl 50uM EDTA and heated at 65°C for 10 minutes. Finally, we used the RNA Clean & Concentrator kit (Zymo Research) following the manufacturer instructions and eluting the samples in 60ul of H_20.

For the RNA extractions from male samples, we used the PureLink RNA Mini Kit (ThermoFisher Scientific), using a slightly modified protocol. We first briefly rinsed the samples in 1X PBS, then added 50μl Trizol into each sample and crushed the tissue with a micropestle. We then added 350μl of Trizol, briefly microcentrifuged the tubes, and transferred the supernatant to a clean tube. We added 80μl of BCP (1-Bromo-3-chloropropane), shook the sample by hand for 15 sec and incubated on ice for 3 min. We then centrifuged the samples at 4°C for 15 min (13,000rpm for all centrifugation steps). We transferred the supernatant to a new tube and added an equal volume of freshly prepared EtOH, mixing the tube by vortexing. We transferred the supernatant to a spin column and centrifuged the sample for 30 sec. We discarded the flow through and added 350μl of Wash Buffer I to the sample. We centrifuged for 30 sec, then added 80ul of the DNase mixture (made of 8μl 10X DNase I reaction buffer, 10μl resuspend DNase I, and 62μl of RNase-free water) onto the membrane of the spin column. We incubated this mixture for 15 min at room temperature, then added 350μl of Wash Buffer I to the sample and centrifuged for 30sec. We then placed the spin cartridge into a new collection tube, added 500μl of Wash Buffer II, and centrifuged the sample for 30sec. We repeated this step once, then centrifuged the sample for an extra minute to dry the membrane. We placed the spin cartridge into a clean eppendorf tube, added 30μl RNase-free water to the membrane, and incubated the sample for 1min. We centrifuge the sample for 2 min and stored the sample at -80°C. We performed a cDNA amplification of the male samples as the yield of RNA from these samples was small

due to their small size. In order to do this, we used the Ovation RNAseq System V2 (Tecan), following the manufacturer protocol.

Supplementary Tables/ Figures

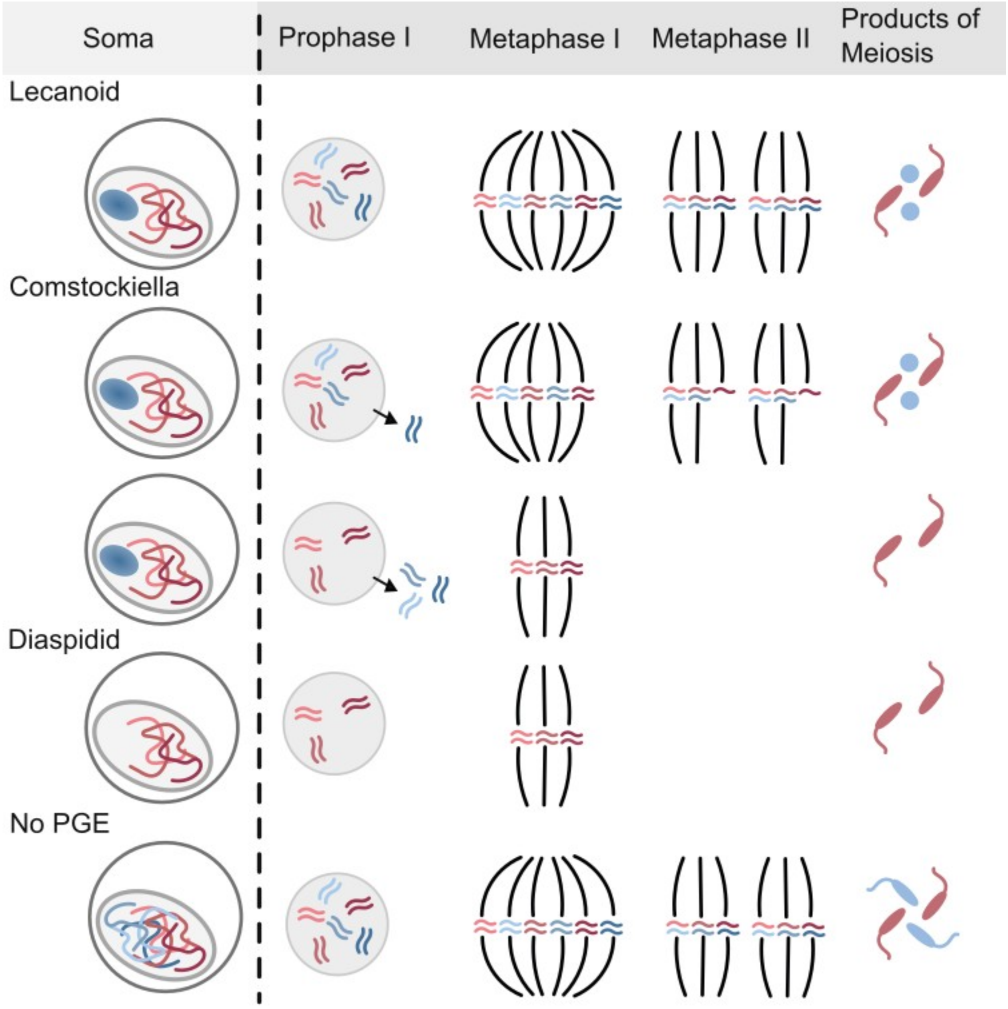

Supplementary Figure 1. Differences in the mechanism of PGE in scale insect species. All scale insects exhibit inverted meiosis (i.e. sister chromatids separate in the first division of meiosis while homologous chromosomes separate in the second division) and chromosomes segregate according to their parent of origin. There are three types of PGE in scale insects; Lecanoid, Comstockiella, and Diaspidid. In the Lecanoid system, paternally inherited chromosomes (blue, maternal chromosomes in red) are retained in males throughout development, and are condensed as heterochromatic bodies in somatic cells (blue circle representing the condensed paternal chromosomes in a ball). During meiosis, paternal chromosomes are present throughout meiosis, but form into non-viable pycnotic nuclei (blue circles), which are degraded following meiosis. In the Comstockiella system, male somatic cells look similar to Lecanoid species, but some paternal chromosomes are eliminated from cells just prior to meiosis. The number eliminated is variable between/within species, and pycnotic nuclei can be either present or absent depending on the number eliminated. In the Diaspidid system, paternally inherited chromosomes are eliminated from all cells of males in early development, so heterochromatic bodies are absent from the male

soma and pycnotic nuclei are not present after meiosis, which has only one division (as there are no paternal chromosomes). Finally, the last row represents a hypothetical scenario of what somatic cells and meiosis might look like in a species which lacks PGE transmission but evolved from an ancestor with this type of reproduction. In this case, the paternally inherited chromosomes form into viable sperm (i.e. no pycnotic nuclei) and there are two divisions in meiosis and no heterochromatic bodies in somatic cells.

Supplementary Table 1. Sample collection Information for samples used in this study.

Species	Purpose	Collection date	Sample ID	Collection location	Latitude	Longitude	Collector
Cystococcus campanidorsalis	Microsatellite	2013	TLS_080F1		-27.45281	152.23029	TLS
	Microsatellite	2013	TLS_081F2		-27.45281	152.23029	TLS
	Microsatellite	2013	TLS_091F1		-24.39534	151.04507	TLS
	Staining, microsatellite	2013	TLS_091F2		-24.39534	151.04507	TLS
	Staining, microsatellite	2013	TLS_095F1		-24.4501	150.94395	TLS
	Phylogeny	26.iv.2008	LGC_00847	Crows Nest National Park, Qld	-27.2599	152.1172	LGC
	Microsatellite	14.xii.2009	LGC_01363	Giles Rd, Redland Bay, Qld	-27.3652	153.1656	LGC
	Staining, microsatellite	23.ii.2014	LGC_02450F2	Allies Creek State Forest, Qld	-25.95579	151.202625	LGC
	RNAseq	7.v.2017	LGC_03538	Lockyer Nat. Park, Qld	-27.4791	152.28159	CNH
Cystococcus echiniformis	Microsatellite	19.x.2013	TLS_064		-27.72208	142.82253	TLS
	Staining, microsatellite	19.x.2013	TLS_069	39km W of Thargomindah, QLD	-27.81193	143.51685	TLS
	Microsatellite	2013	TLS_087F1		-24.58807	148.88364	TLS
	Staining, microsatellite	2013	TLS_100		-26.07543	152.39366	TLS
	Microsatellite	12.ix.2006	LGC_00628	12 km W of Herberton, Qld	-17.2248	145.1816	LGC
	Phylogeny, microsatellite	1.x.2009	LGC_01267	Town Lookout near Timber Creek, NT	-15.3846	130.2733	LGC
	Microsatellite	3.x.2009	LGC_01272	Keep River National Park, NT	-15.4501	129.0509	LGC
	Microsatellite	1.x.2009	LGC_01288	Joe Creek, Gregory National Park, NT	-15.3625	131.0442	LGC
	Staining, microsatellite	30.viii.2014	LGC_02523F2	Valley of Lagoons Rd, Qld	-18.515833	144.7836	LGC
	Staining, microsatellite	31.viii.2014	LGC_02525	Einasleight-Forsayth Rd, Qld	-18.54877	143.94232	LGC
	Microsatellite	20.ix.2014	LGC_02600	Homevale NP, Qld	-21.4078	148.5058	LGC
	Microsatellite, RNAseq	28.v.2017	LGC_03571F4	Hawkwood Rd, SW of Munduberra, Qld	-25.6956	150.9733	CNH
	Microsatellite, RNAseq	28.v.2017	LGC_03571F5	Hawkwood Rd, SW of Munduberra, Qld	-25.6956	150.9733	CNH

	RNAseq	29.v.2017	LGC_03572F4	Burnett Hwy, E of Gayndah, Qld	-25.6113	151.662	CNH
Cystococcus pomiformis	Staining	30.viii.2014	LGC_02534	Gregory Developmental Rd, Qld	-18.9961	144.695	LGC
	Staining	31.viii.2014	LGC_02530	Georgetown-Mt Garnet Rd, Qld	-18.8494	144.4247	LGC
	Staining	2.ix.2014	LGC_02536	Einasleigh-Forsayth Rd, Qld	-18.5447	143.8775	LGC
	Phylogeny, Staining	18.ix.2004	LGC_00267	Palm Grove Caravan Park, WA			LGC&MDC
Ascelis praemollis	Staining	17.ix.2014	LGC_02583	Wild Rivers Caravan Park, Herberton	-17.22	145.2318	LGC
	Staining	14.x.2015	LGC_02899	60 Bretons Rd, Crohamhurst, Qld	-26.802	152.87	LGC
	Phylogeny	16.vii.1994	Asc1	391 Fisherman's Reach Rd, Stuarts Point	-30.49	153	LGC
Ascelis schraderi	Staining		PJM_00515	School Road, Yeerongpilly, Qld	-27.521323	153.021189	PJM
	Phylogeny	26.xii.2006	LGC_00718	nr Trial Bay, South West Rocks, NSW	-30.52	153.03	LGC
Callococcus acaciae	Staining	4.x.2014	LGC_02618	Ku-ring-gai Chase NP, NSW	-33.673	151.134	LGC
	Staining	3.x.2014	LGC_02614	Red Hill reserve, Oxford Falls, NSW	-33.741	151.253	LGC
	Phylogeny	1.xi.2007	LGC_01124	O'Connor, ACT			MDC
Callococcus leptospermi	Staining	4.xi.2014	LGC_02626	Myall Lakes NP, NSW	-32.5	152.285	LGC
	Staining	3.x.2014	LGC_02612	Elvina walking track carpark, NSW	-33.643	151.262	LGC
	Phylogeny, Staining	24.xii.2016	LGC_03410	St John's Wood Rd, Blairgowrie, Vic			LGC
Cylindrococcus sp.	Staining	2017	LGC_01956	Burrabaranga Rd, Durikai State Forest, Qld	-28.26589	151.54376	GPH
	Staining		Fresh	Brisbane			LGC
	Phylogeny	26.ix.2006	LGC_00657	Heathlands Resource Reserve, Qld	-11.38066	142.44216	LGC
Eriococcus coriaceus	Staining	1.vii.2014	LGC_02489	Bellangry State Forest, NSW	-31.288056	152.508611	LGC

	Staining	v.2017	Fresh	Colony on UQ, St. Lucia Campus, Qld			CNH
Sphaerococcus ferrugineus	Staining	13.ix.2014	LGC_02572	Mt Emerald, near Tolga, Qld	-17.2103	145.4326	MDC
	Phylogeny	16.x.2006	LGC_00685	UQ, St Lucia Campus, Qld	-27.5005767	153.0152758	LGC
Sphaerococcus socialis	Staining	5.x.2010	LGC_01654	Chester Pass Rd, WA	-34.562166	118.00819	MDC
Opisthoscelis subrotunda	Staining	12.vi.2010	LGC_01426	Mt Tibrogargan, Qld	-26.55435	152.5652	LGC
	Phylogeny	8.ii.2004	LGC_00099	Midland Hwy, c. 27 km E of Shepparton, Vic	-36.26	145.42	PJG
Tanyscelis sp.	Staining	16.ix.2014	LGC_02577	Herberton-Irvinebank Rd, Qld	-17.38856	145.34669	LGC
Tanyscelis convexa	Phylogeny	17.x.2003	LGC_00043	Kennedy Hwy, 45 km SW of Mt Garnet, Qld	-16.5805	144.5144	LGC&MDC

Supplementary Table 2. Summary of samples stained and the results of cell staining for somatic cells as well as male tissue undergoing meiosis. For *Tanyscelis sp.* and *Capulinia jaboticabae,* we used sequence from a related species in the same genus in the phylogeny. We got the sequence for *Parasaissetia nigra* from NCBI (Accession: KY927598.1, KY924795.1). HB = heterochromatic body.

Species	Reference	#	Life stage/ sex examined	Somatic HB	Meiosis stages examined	Number cells/ sperm cyst	Number sperm/ bundle	Pycnotic nuclei	Loci for phylogeny
Cystococcus campanidorsalis	this study	20	male nymphs/ pupae	Yes	all	16	32	No	18S, COI
Cystococcus pomiformis	this study	30	male nymphs/pupae/ adults	Yes	Prophase/ sperm bundles	8 or 16	16 or 32	No	18S, COI
Cystococcus echiniformis	this study	25	male nymph/ pupae/ adults	No	all	8 or 16	16 or 32	No	18S, COI
Ascelis praemollis	this study	6	male nymphs/pupae	No	all	8	16	No/ few	18S, CO1
Ascelis schraderi	this study, Brown 1967	8	male nymphs/pupae	Yes	Prophase/ metaphase	8	16	No	18S, COI
Callococcus acaciae	this study	11	mixed sex crawlers/ early male nymphs	No					18S, COI
Callococcus leptospermi	this study	11	mixed sex crawlers/ early male nymphs	Yes					18S, COI
Cylindrococcus sp.	this study	22	mixed sex crawlers/ male nymphs and adults	Yes					18S, COI
Eriococcus coriaceus	this study, Brown 1967	16	mised sex crawlers/ male pupae	Yes	Sperm bundles			Yes	18S, COI
Sphaerococcus ferrugineus	this study	3	male nymphs/ pupae	Yes	Sperm bundles			Yes	18S, COI
Sphaerococcus socialis	this study	11	mixed sex crawlers/ male nymphs	Yes					18S, COI
Opisthoscelis subrotunda	this study	32	mixed sex eggs/ crawlers/ male pupae	Yes					18S, COI
Tanyscelis sp.	this study		male nymphs	Yes					18S, COI
Apiomorpha pharetrata	Brown, 1967			Yes					18S
Apiomorpha calycina	Brown, 1967			Yes					18S, COI
Apiomorpha (45 species)	Cook, 2000; 2001			Yes					
Capulinia jaboticabae	Brown, 1967			Yes					18S, COI

Capulinia orbiculata	Brown, 1967	Yes	
Casuarinaloma leaii	Brown, 1967	Yes	
Cylindrococcus spiniferus	Brown, 1967	Yes	
Eriococcus abditus	Brown, 1967	Yes	
Eriococcus araucariae	Brown, 1967	Yes	18S, COI
Eriococcus detectus	Brown, 1967	Yes	
Eriococcus lecanioides	Brown, 1967	Yes	
Eriococcus mimus	Brown, 1967	Yes	
Eriococcus rata	Brown, 1967	Yes	
Eriococcus rhodomyrti	Brown, 1967	Yes	
Lachnodius eucalypti	Brown, 1977	No	18S, COI
Eriococcus leptospermi	Brown, 1967	Yes	18S, COI
Madarococcus cavellii	Brown, 1967	Yes	18S
Madarococcus totarae	Brown, 1967	Yes	18S
Madarococcus viridulus	Brown, 1967	Yes	18S
Ourococcus cobbi	Brown, 1967	Yes	18S
Phloeococcus loriceus	Brown, 1967	Yes	
Tanyscelis convexa	Brown, 1967	only in germ tissue	
Stictococcus sp.	Brown, 1977	No	18S
Parasaissetia nigra (outgroup)		Partheno-genetic	18S,CO1

Supplementary Table 3. Accession numbers for sequences used in figure 2.

Species	18S	CO1
Cystococcus campanidorsalis	KP729358.1	KP729338.1
Cystococcus echiniformis	OX461210	OX463790
Cystococcus pomiformis	OX461211	OX463791
Ascelis praemollis	AY795523.1	OX463796
Ascelis schraderi	KP729354.1	KP729334.1
Callococcus acaciae	OX461212	OX463797
Callococcus leptospermi	OX461213	OX463792
Sphaerococcus "Beesonia" ferrugineus	OX461216	OX463799
Sphaerococcus socialis	OX461217	OX463794
Capulinia sp.	OX461222	OX463803
Opisthoscelis subrotunda	OX461218	OX463800
Lachnodius eucalypti	OX461225	OX463804
Tanyscelis sp.	OX461219	OX463801
Madarococcus cavellii	OX461224	NA
Madarococcus totarae	AY795534.1	NA
Madarococcus viridulus	AY795529.1	NA
Cylindrococcus spiniferus	OX461214	OX463798
Stictococcus sp.	AY795509.1	NA
Apiomorpha pharetrata	OX461221	NA
Apiomorpha calycina	OX461220	OX463802
Eriococcus coriaceus	OX461215	OX463793
Eriococcus leptospermi	AY795546.1	OX463795
Ourococcus cobbi	AY795550.1	NA
Eriococcus araucariae	OX461223	NA
Parasaisettia nigra	KY927598.1	KY924795.1

Supplementary Table 4. Primer information for 18S and cytochrome oxidase I (COI) loci sequenced for the phylogeny in Figure 2. The same primer set was used for all species for the 18S loci, while for most species for the CO1 loci, the PCO_F1/HCO primer set was used with the exception of *Cystococccus* and *Ascelis* species, for which the CystoCOIF/CystCOIR primer set was used.

Locus	Primer name	Primer Sequence	Program	Reference
18S	2880 (F)	GTTTTCCCAGTCACGACCTGGT TGATCCTGCCAGTAG	94°C/3:00, 34x (94°C/0:30, 55°C/0:30, 72°C/1:00), 72°C/5:00	Tautz et al. 1988; von Dohlen and Moran 1995
	Br (R)	CCGCGGCTGCTGGCACCAGA		
COI	PCO_F1(F)	CCTTCAACTAATCATAAAAATATY AG	95°C/2:00, 5x (94°C/0:40, 72°C/1:10), 40x (94°C/0:40, 51°C/0:40, 72°C/1:10), 72°C/10:00	Park et al. 2010;
	HCO (R)	TAAACTTCAGGGTGACCAAAAAA TCA		
COI	CystCOIF (F)	TGRTCAGGAATAATAGGAATA	95°C/2:00, 5x (94°C/0:40, 72°C/1:10), 40x (94°C/0:40, 51°C/0:40, 72°C/1:10), 72°C/10:00	Semple et al., 2015
	CystCOIR (R)	GTATTYAAAAATCTTGTTGATATG TT		

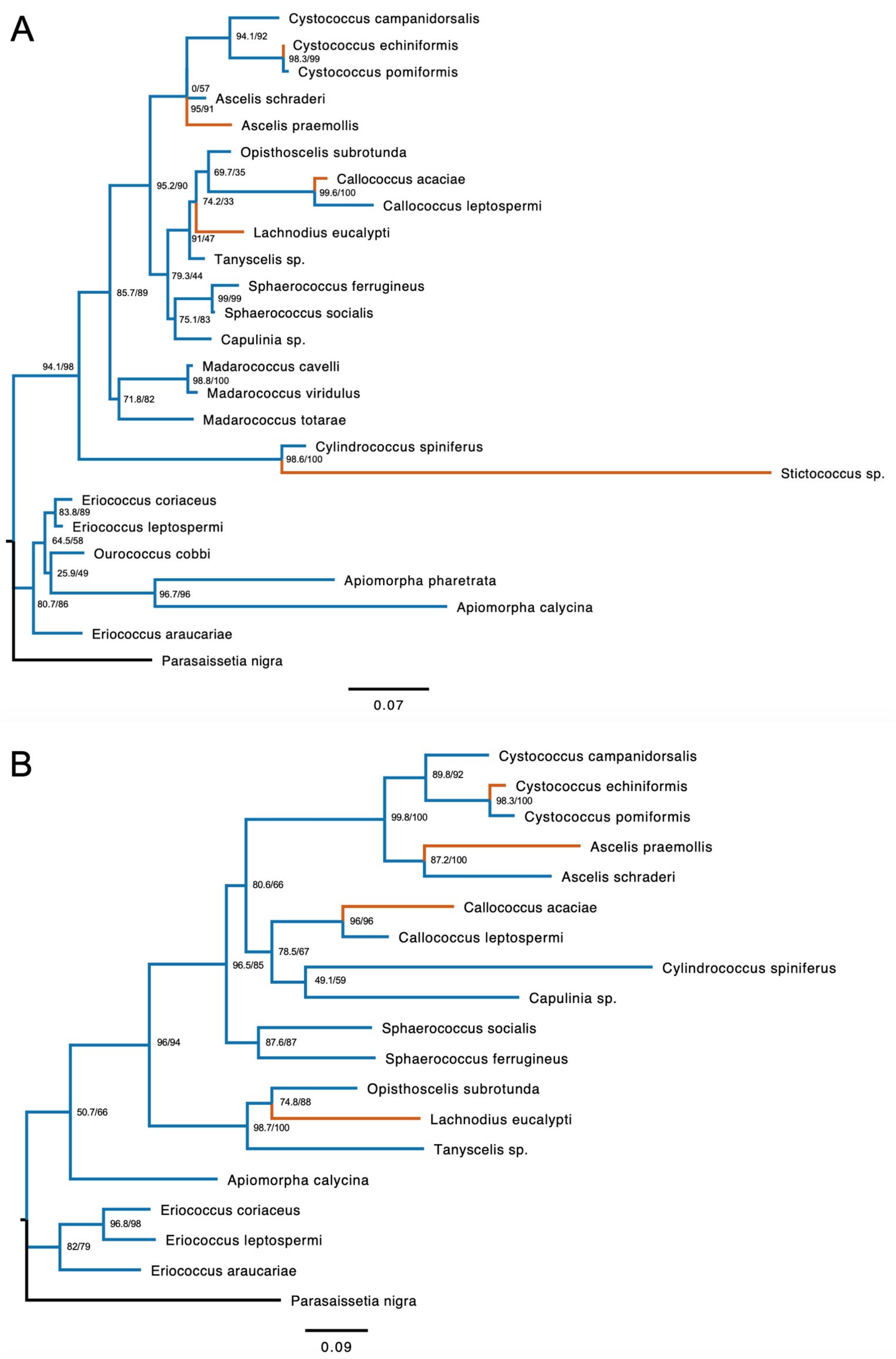

Supplementary Figure 2. Phylogeny of Eriococcidae species using an approximately 650 bp region of the nuclear gene 18S (**A**), and an approximately 600bp region of the mitochondrial gene CO1 (**B**). Several species have only 18S sequence available (Supplementary Table 3), and so are not included in (**B**).

Supplementary Table 5. Primer information for the microsatellite inheritance study, including primer sequences, the expected size of the PCR product for each primer pair, and which PCR panel each primer set belonged to. The M13 fluorescent primer was added into all PCR reaction mixes with a fluorescent tag to fluorescently label PCR products.

Species	Primer name	Forward primer sequence	Reverse primer sequence	Expected size	PCR panel
C. campanidorsalis	cyst_msat_08	GTAAAACGACGGCCAGAATTTCCATTCGGTAGTAGG	CAGTACAGTTACCATTCCACAG	150	1
	cyst_msat_09	GTAAAACGACGGCCAGAAGGGAATTTAATGGTATGC	GAGGTTGGTGGTTCTAAGTG	150	2
	cyst_msat_12	GTAAAACGACGGCCAGCGATGCGTTGAATATTAGTG	ATGTTCCGTGACCTAACTTG	194	3
	cyst_msat_14	GTAAAACGACGGCCAGGAACCAAGCCAATAATGATC	GCAGCCCAAATATGAAGAG	203	1
	cyst_msat_16	GTAAAACGACGGCCAGAAAGGTTGAAGGGTAGTGG	AACGGGAAATGTAAATTGAG	217	1
	cyst_msat_17	GTAAAACGACGGCCAGAAATTATGGCCTTGAGTTG	TTAGGTGCCTCATACGTCAG	221	3
	cyst_msat_18	GTAAAACGACGGCCAGTTGAGTGCGTAATTGAATTG	CACAGGCAGGTCTCTTAAAG	222	2
	cyst_msat_21	GTAAAACGACGGCCAGGGAAGTGAATTTCGACGTAG	TTGCCCACCTACAGTAGTTC	261	3
C. echiniformis	c.ech_msat77	GTAAAACGACGGCCAGCCCTATACCACGTTCGACCA	CCCTATACCACGTTCGACCA	152	4
	c.ech_msat79	GTAAAACGACGGCCAGAAGCGTGTCTTCGTCTCGAT	AAGCGTGTCTTCGTCTCGAT	124	5
	c.ech_msat80	GTAAAACGACGGCCAGTATTGCGCTATCTCATCGGA	TATTGCGCTATCTCATCGGA	175	6
	c.ech_msat89	GTAAAACGACGGCCAGTTGACAAGAGCGAGAATTTCC	TTGACAAGAGCGAGAATTTCC	238	4
	c.ech_msat61	GTAAAACGACGGCCAGGTCGTTAACCGATGGCAGAC	GTCGTTAACCGATGGCAGAC	206	6
	c.ech_msat97	GTAAAACGACGGCCAGGGAATTCTATGCGAGGTTGC	GGAATTCTATGCGAGGTTGC	234	6
	c.ech_msat99	GTAAAACGACGGCCAGCCACACCTCTTCGAAAGTCC	CCACACCTCTTCGAAAGTCC	197	4
	c.ech_msat102	GTAAAACGACGGCCAGGGTCTTCCACGGATCAGTAGTT	GGTCTTCCACGGATCAGTAGTT	141	5
	c.ech_msat104	GTAAAACGACGGCCAGGGGAGTCTTTACCACCTACGA	GGGAGTCTTTACCACCTACGAT	233	5

	T	
M13 Fluorescent tail	GTAAAACGACGGCCAG	N/A

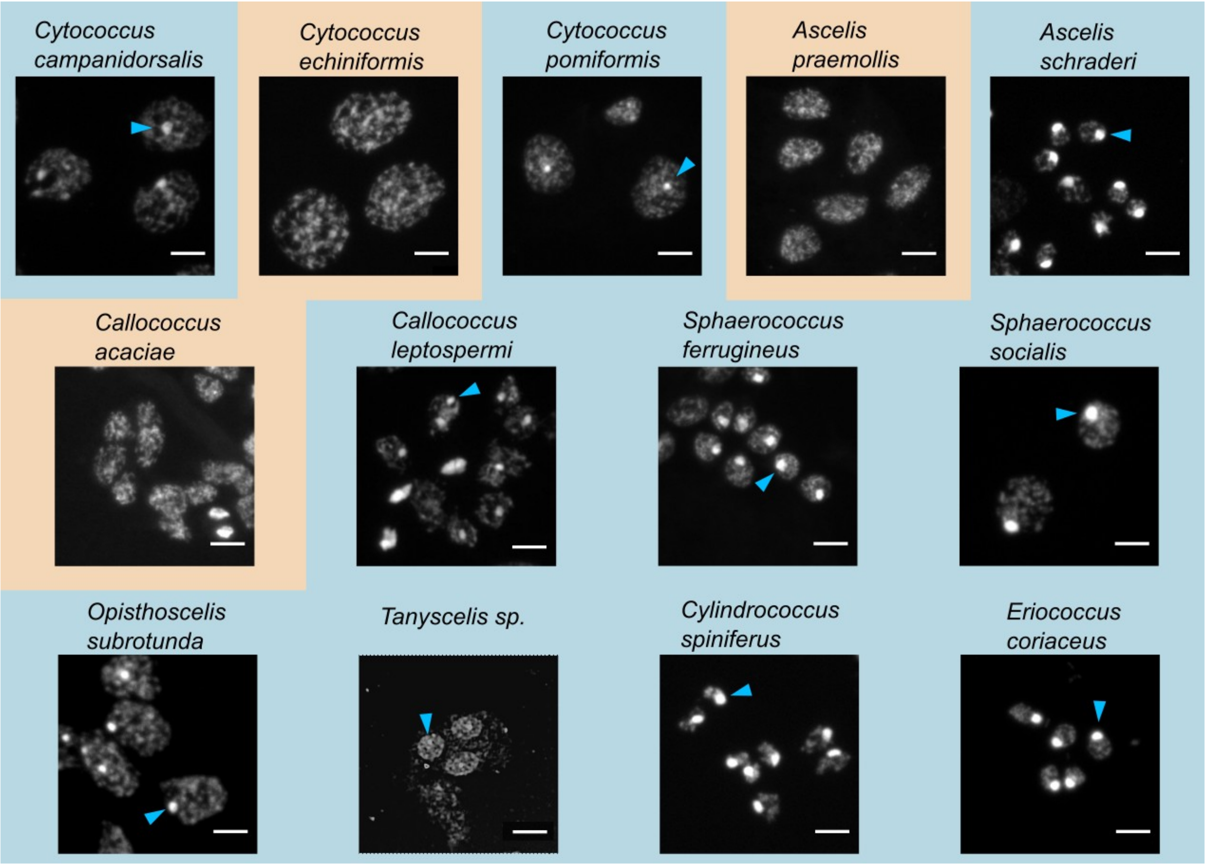

Supplementary Figure 3. Male somatic cells for all Eriococcidae species investigated in this study. Most species (blue background) have heterochromatic bodies (indicated with blue arrows) in somatic cells, which are brightly staining bodies containing condensed paternally inherited chromosomes. As heterochromatic bodies is a hallmark of PGE in scale insects, we presume these species have PGE. Three species (orange background) lack heterochromatic bodies in somatic cells, suggesting they either lack PGE, or have a different type of PGE to other Eriococcidae species.

Supplementary Table 6: BUSCO results for de novo transcriptomes for *Cystococcus campanidorsalis* and *C. echiniformis*. Both species have more than 95% of the single copy orthologs expected to be present in insect genomes.

	Cystococcus campanidorsalis		*Cystococcus echiniformis*	
Complete	1591	95.90%	1589	95.80%
Single copy	95	5.70%	113	6.80%
Duplicated	1496	90.20%	1476	89.00%
Fragmented	21	1.30%	21	1.30%
Missing	46	2.80%	48	2.90%
Total	1658		1658	

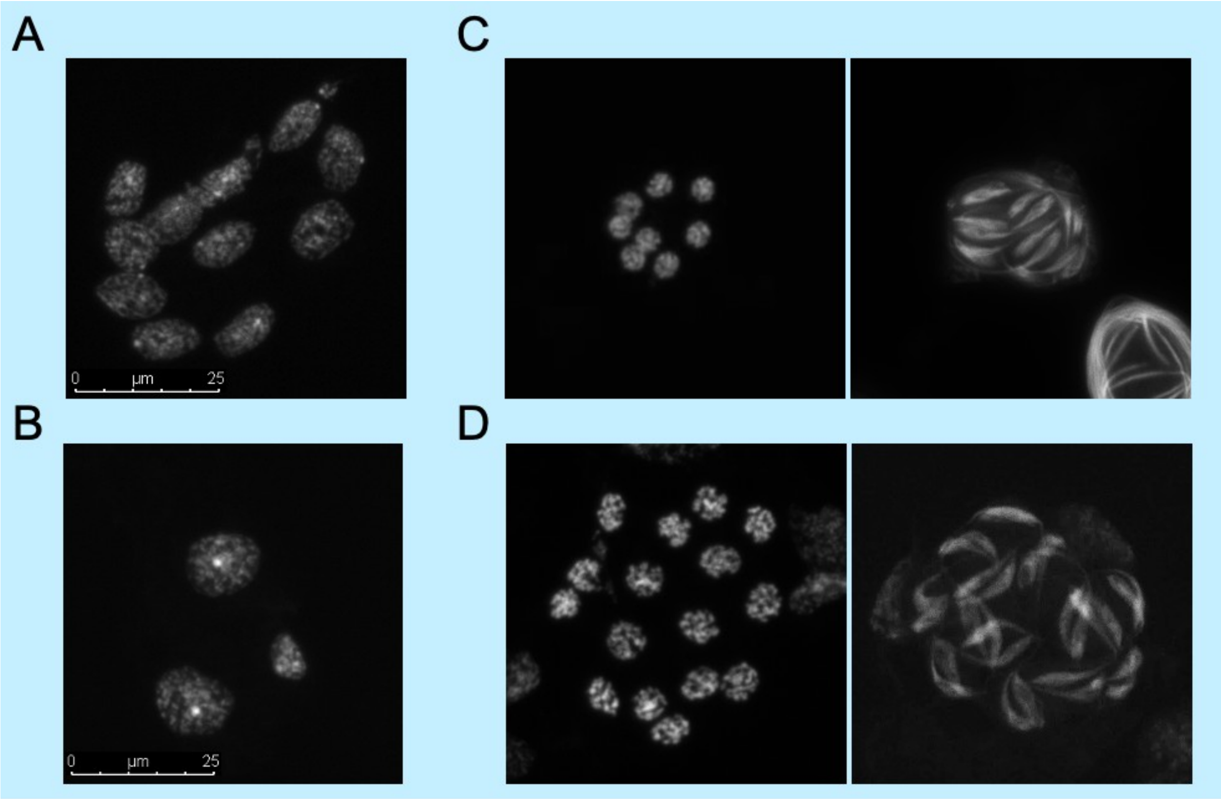

Supplementary Figure 4. Meiosis in *C. pomiformis*. (**A)** and **(B)** show somatic cells, (**A)** without (or with small) heterochromatic bodies and (**B)** with clear small heterochromatic bodies. (**C)** shows a sperm cyst with 8 nuclei and 16 sperm at the end of meiosis, while (**D)** shows a sperm cyst with 16 nuclei and 32 sperm at the end of meiosis. *Cystococcus pomiformis* likely exhibits Comstockiella PGE with only one division in meiosis, but sperm cysts can differ in how many nuclei are present at the beginning of meiosis.

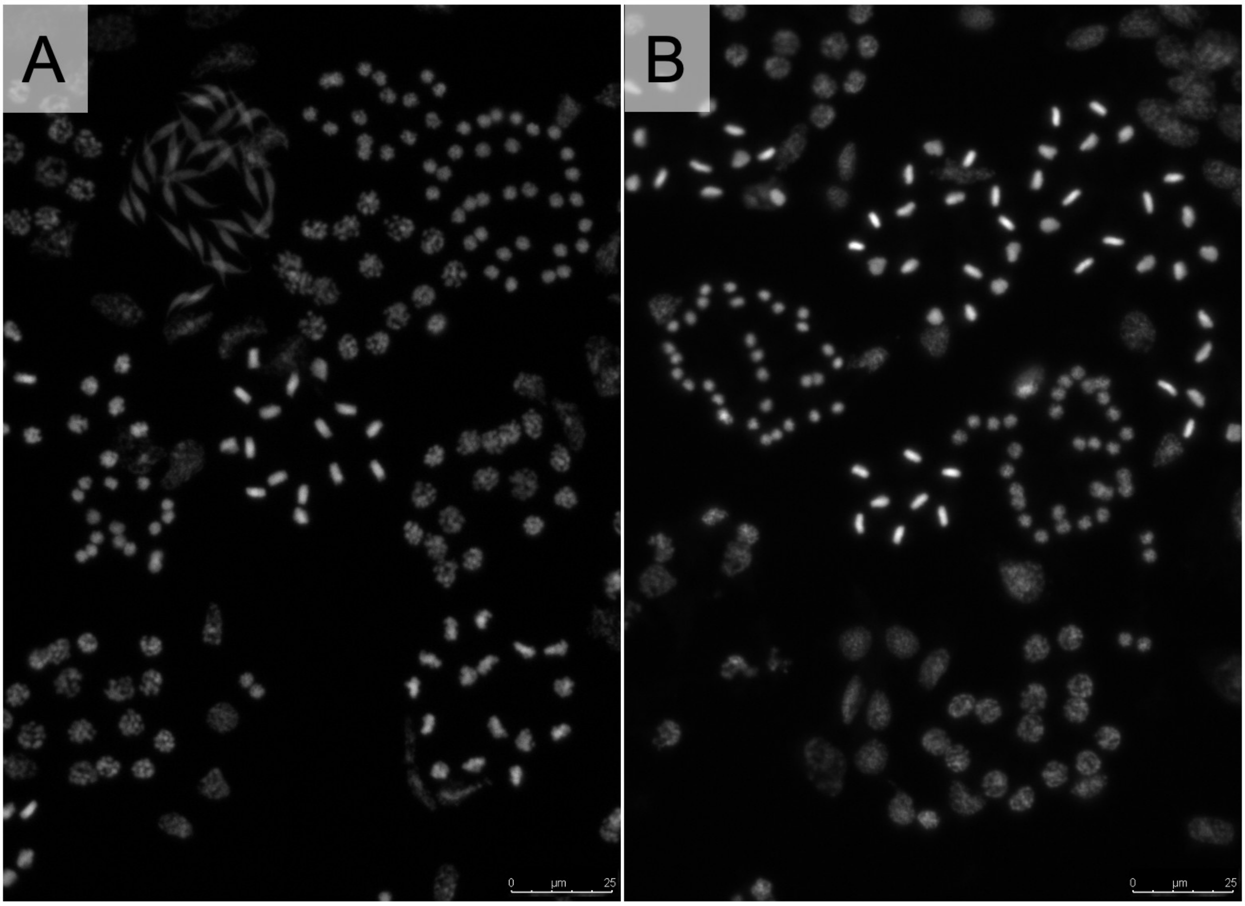

Supplementary Figure 5. Sperm cysts undergoing meiosis in *Cystococcus campanidorsalis* (**A**) and *C. echiniformis* (**B**). Sperm cysts in *C. echiniformis* can have either 8 or 16 primary spermatids in each sperm cyst, (both pictured) but cysts with 16 primary spermatids are more common.

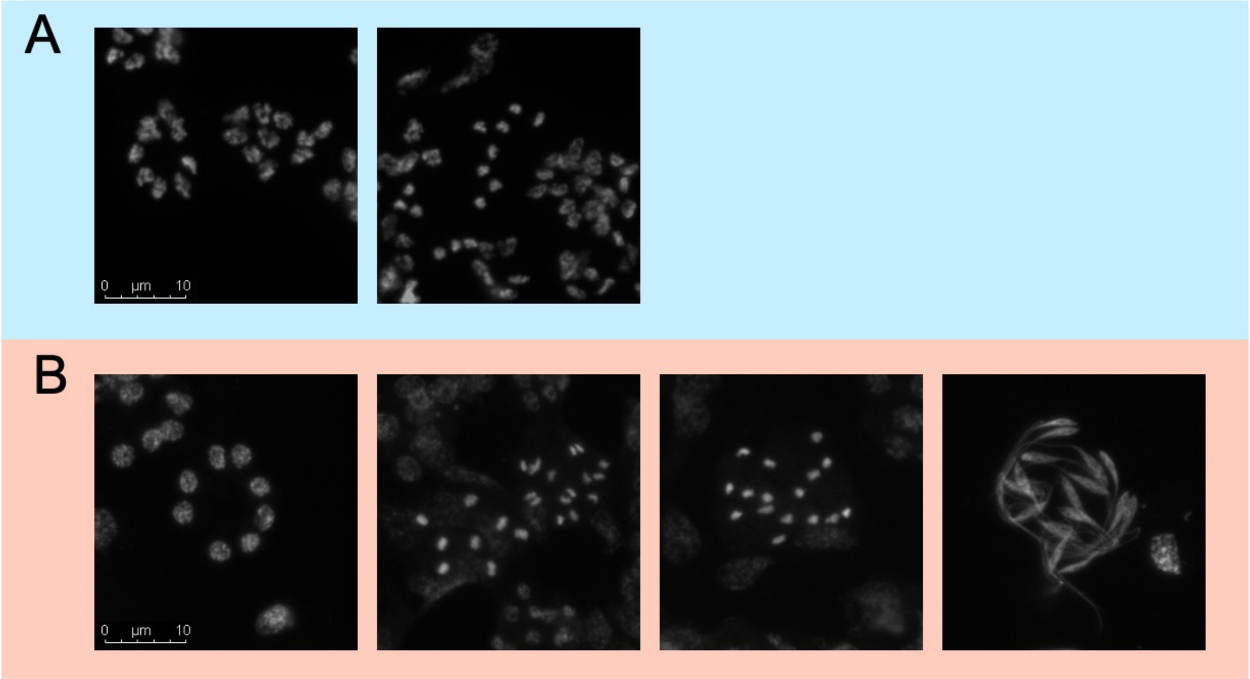

Supplementary Figure 6. Male meiosis in *Ascelis schraderi* (**A**) and *A. praemollis* (**B**). In both species male meiosis has one division with 8 nuclei in each sperm cyst at the beginning of meiosis and 16 sperm in each sperm bundle (information for *A. schraderi* from Brown, 1967). This indicates that both species have Comstockiella PGE.

Supplementary Table 7. Summary of the count of the number of nuclei in each sperm cyst/sperm bundle in *C. echiniformis* and *C. pomiformis* slides.

Species	Sample Location	Slide Number	Stage of meiosis	Count of Nuclei		
				**8**	**16**	**32**
C. echiniformis	TLS69	1	Cyst with nuclei in prophase	36	52	
	TLS69	2	Cyst with nuclei in prophase	7	16	
	TLS100	1	Cyst with nuclei in prophase			
	LGC2525	1	Sperm bundles forming		1	2
	LGC2525	2	Sperm bundles forming		1	0
	TLS69	3	Cysts with nuclei going through meiosis (prophase, metaphase, anaphase, sperm bundles forming)	12	62	23
C. pomiformis	TLS_001	1	Cyst with nuclei in prophase	1	4	
	TLS_001	2	Cyst with nuclei in prophase	6	20	
	TLS_001	3	Cyst with nuclei in prophase	3	16	
	TLS_001	4	Cyst with nuclei in prophase	1	8	
	LGC2536	1	Sperm bundles forming		11	44

* if there is one division in meiosis 8 nuclei cysts will give rise to sperm bundles with 16 sperm, while 16 nulcei cysts will give rise to sperm bundles with 32 sperm.

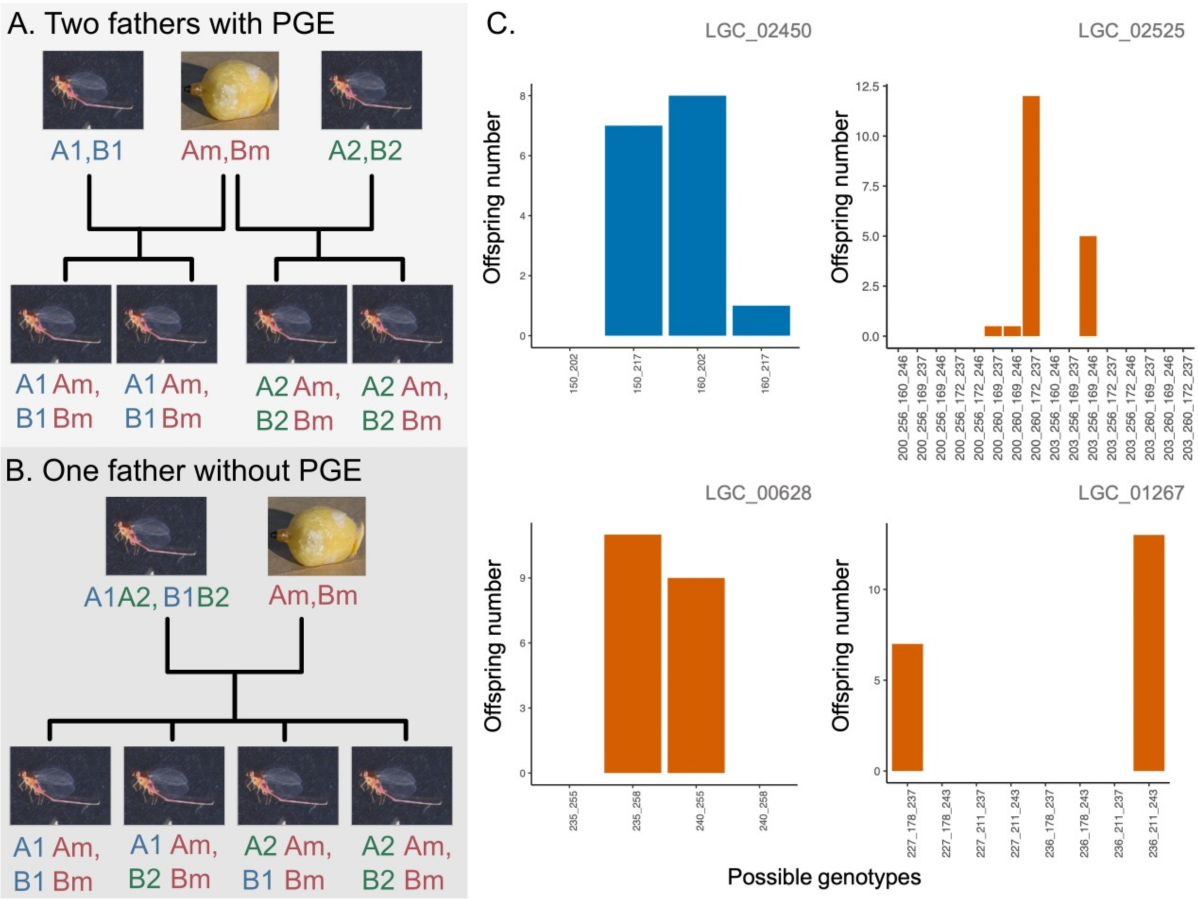

Supplementary Figure 7. (**A-B**) Schematic showing how *Cystococcus* families may have inherited more than one allele from their father. In (**A**) two fathers that exhibit PGE mated with the focal female (Am, Bm), producing two sons with the genotype of the first male, and two sons with the genotype of the second male (for both microsatellite loci A and B), while in (**B**), the female mated with one male who does not exhibit PGE and passes the two alleles for A and B to his offspring randomly. The sons in this scenario are expected to have all possible combination of alleles. (**C**) Number of offspring with each allele combination in *Cystococcus campanidorsalis* (blue) and *C. echiniformis* (orange) families in which sons inherited two paternal alleles for two or more microsatellite loci. In all four families, not all allele combinations are present that would be expected if males had one father that was transmitting alleles in a Mendelian fashion. Rather, allele inheritance patterns are more similar to scenario (**A**). The values on the x-axis indicate the size of each PCR amplicon for each genotype.

Supplementary Table 8: Model output analysing whether families inherit a different number of alleles depending on whether the allele is of maternal or paternal origin and whether the males are *C. echiniformis* or *C. campanidorsalis* males. The estimate and the standard error (in parentheses) is shown as well as whether the factor was predicted to be significant.

	Number of alleles inherited
Species	0.627(0.483)
Parent	2.382 (0.503)***
Constant	-0.342(0.438)
Observations	38
Log Likelihood	-65.297
AIC	138.595
BIC	145.145

Note: *p<0.05, **p<0.01, ***p<0.001

Supplementary Table 9. In the four cases in which families inherited two paternal alleles for more than one microsatellite loci (one for *C. campanidorsalis*- LGC02450, and three for *C. echiniformis*), analysis of whether the inheritance patterns of alleles from the two or more sets of microsatellite loci were random. In all cases, Chi-square tests suggest that the allele inheritance is not random, likely because fathers of these families do exhibit PGE, but that the mothers of these families mated with more than one male. Family LGC_02450: 2 loci, LGC_00628: 2 loci, LGC_01267: 3 loci, LGC_02525: 4 loci.

	LGC_02450	LGC_00628	LGC_01267	LGC_02525
Chi-square	12.5	20.4	67.2	132.67
d.f.	3	3	7	15
p-value	0.00585	0.00014	<0.0001	<0.0001

Supplementary Table 10: Summary of number of homozygous (Hom) and heterozygous (Het) SNPs called for each sample in the RNAseq study analysing whether *C. campanidorsalis* and *C. echiniformis* males only express maternally inherited alleles. We performed Fishers exact tests comparing the number of homozygous and heterozygous alleles expressed in sons compared to their mother, although these tests generally suggest that the number of heterozygous SNPs was different between mothers and sons, both family members exhibited significant heterozygous expression of alleles for both species, with between 59-85% of alleles called as heterozygous.

Species	Family	Individual	# SNP (after filtering)	# Hom SNP	# Het SNP	Total SNP in analysis	% Hom SNP	% Het SNP	Fishers test (p-value)	odds ratio
C. echiniformis	LGC_03572F4	Female	11803	3939	6870	10809	36.44%	63.56%		
		Male1	14312	4792	8046	12838	37.33%	62.67%	0.0007333	1.114
		Male2	14202	4095	8073	12168	33.65%	66.35%	2.91E-10	1.201
	LGC_03571F6	Female	10681	3596	6220	9816	36.63%	63.37%		
		Male1	11624	4159	5989	10148	40.98%	59.02%	2.91E-10	1.201
		Male2	8155	2824	4383	7207	39.18%	60.82%	0.0007333	1.114
	LGC_03571F5	Female	12679	4398	7244	11642	37.78%	62.22%		
		Male1	15191	5398	8255	13653	39.54%	60.46%	0.004216	1.077
		Male2	13271	4523	7371	11894	38.03%	61.97%	0.6968	1.011
C. campanidorsalis	LGC_03538F4	Female	25398	4961	17492	22453	22.10%	77.90%		
		Male1	23272	3032	17847	20879	14.52%	85.48%	1.70E-05	0.889
		Male2	26841	3610	21007	24617	14.66%	85.34%	5.31E-05	0.900

Supplementary Figure 8. Image of *Ascelis praemollis* germ tissue, showing paternally inherited chromosomes potentially being eliminated from cells (arrows). As these cells have not yet undergone meiosis, this suggests that chromosome elimination prior to meiosis in this species may be common. However, this image also suggests that different numbers of chromosomes may be eliminated prior to meiosis, as the size of the chromosomes being eliminated varies for different cells.

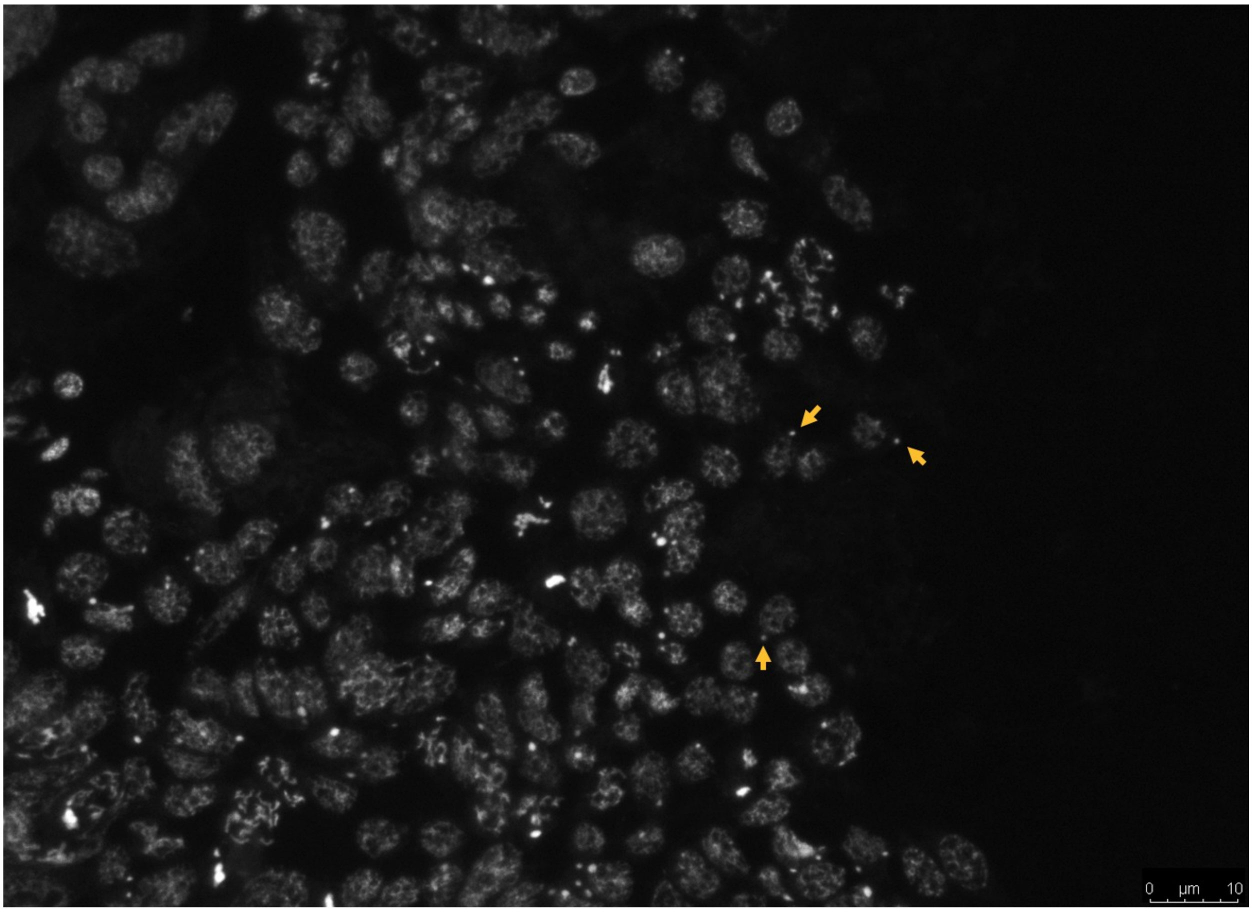

Supplementary references

Chen S, Zhou Y, Chen Y, Gu J (2018). Fastp: An ultra-fast all-in-one FASTQ preprocessor. *Bioinformatics* **34**: i884–i890.

Meglécz E, Costedoat C, Dubut V, Gilles A, Malausa T, Pech N, *et al.* (2009). QDD: A user-friendly program to select microsatellite markers and design primers from large sequencing projects. *Bioinformatics* **26**: 403–404.

Meglecz E, Pech N, Gilles A, Dubut V, Hingamp P, Trilles A, *et al.* (2014). QDD version 3 . 1 : a user-friendly computer program for microsatellite selection and primer design revisited : experimental validation of variables determining genotyping success rate. *Mol Ecol Resour* **14**: 1302–1313.

Schuelke M (2000). An economic method for the fluorescent labeling of PCR fragments. *Nat Biotechnol* **18**: 233–234.

Untergasser A, Cutcutache I, Koressaar T, Ye J, Faircloth BC, Remm M, *et al.* (2012). Primer3-new capabilities and interfaces. *Nucleic Acids Res* **40**: 1–12.
